# The output of the tRNA modification pathways controlled by the *Escherichia coli* MnmEG and MnmC enzymes depends on the growth conditions and the tRNA species

**DOI:** 10.1093/nar/gkt1228

**Published:** 2013-11-26

**Authors:** Ismaïl Moukadiri, M.-José Garzón, Glenn R. Björk, M.-Eugenia Armengod

**Affiliations:** ^1^Laboratory of RNA Modification and Mitochondrial Diseases, Príncipe Felipe Research Center, 46012-Valencia, Spain, ^2^Department of Molecular Biology, Umeå University, S90187, Sweden and ^3^Biomedical Research Networking Centre for Rare Diseases (CIBERER) (node U721), Spain

## Abstract

In *Escherichia coli*, the MnmEG complex modifies transfer RNAs (tRNAs) decoding NNA/NNG codons. MnmEG catalyzes two different modification reactions, which add an aminomethyl (nm) or carboxymethylaminomethyl (cmnm) group to position 5 of the anticodon wobble uridine using ammonium or glycine, respectively. In 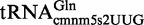 and 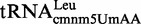, however, cmnm^5^ appears as the final modification, whereas in the remaining tRNAs, the MnmEG products are converted into 5-methylaminomethyl (mnm^5^) through the two-domain, bi-functional enzyme MnmC. MnmC(o) transforms cmnm^5^ into nm^5^, whereas MnmC(m) converts nm^5^ into mnm^5^, thus producing an atypical network of modification pathways. We investigate the activities and tRNA specificity of MnmEG and the MnmC domains, the ability of tRNAs to follow the ammonium or glycine pathway and the effect of *mnmC* mutations on growth. We demonstrate that the two MnmC domains function independently of each other and that 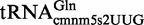 and 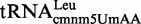 are substrates for MnmC(m), but not MnmC(o). Synthesis of mnm^5^s^2^U by MnmEG-MnmC *in vivo* avoids build-up of intermediates in 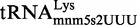. We also show that MnmEG can modify all the tRNAs via the ammonium pathway. Strikingly, the net output of the MnmEG pathways *in vivo* depends on growth conditions and tRNA species. Loss of any MnmC activity has a biological cost under specific conditions.

## INTRODUCTION

Transfer RNAs (tRNAs) are highly and diversely modified, and each has a unique pattern of modification (1–3). These modifications are post-transcriptionally introduced at precise positions by specific enzymes and play important roles in folding, stability, identity and in the functions of tRNAs.

Modifications at the wobble position of the anticodon contribute to the accuracy and efficiency of protein synthesis (1,3,4). Changes in the modification levels of the wobble position affect the synthesis of specific proteins and also lead to complex phenotypes through unknown mechanisms (3,5–10).

Wobble modifications are often complex and require for their synthesis the participation of several enzymes (3). Even though all of the enzymes involved in a specific modification pathway are known, it is unclear how their actions are modulated and coordinated to produce the final modification. Progress in this area may lead to a better understanding of tRNA biology.

**Figure 1.**
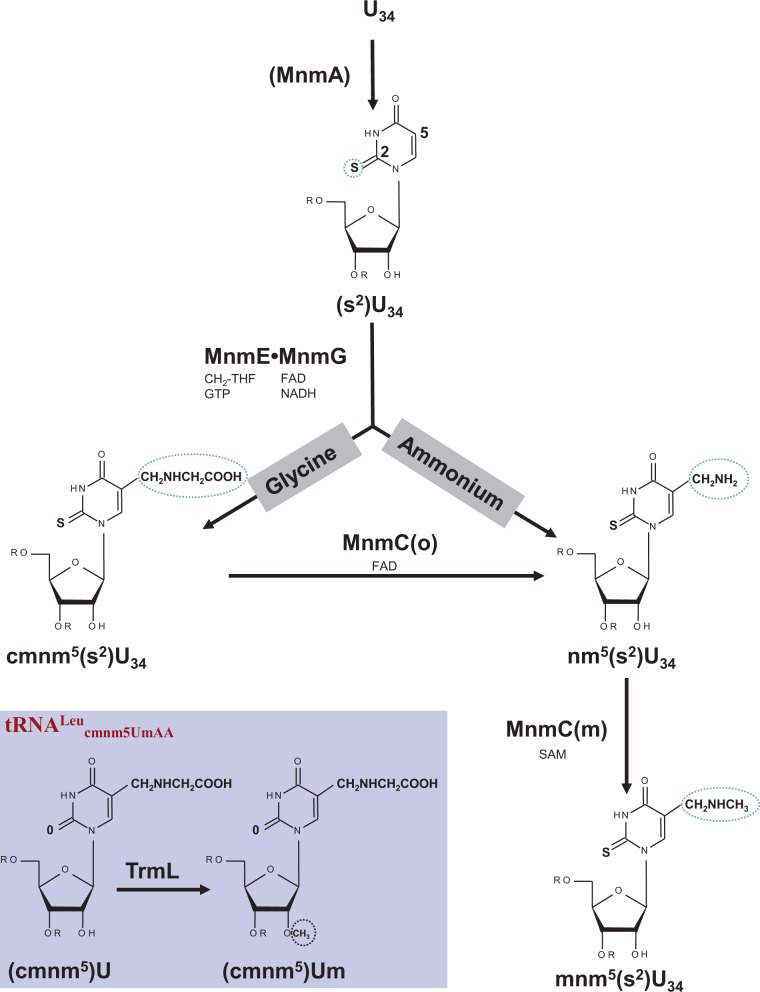
Synthesis of mnm^5^(s^2^)U in *E. coli*. The MnmEG complex formed by proteins MnmE and MnmG is active on position 5 of the wobble uridine (U34) in 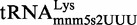, 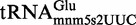, 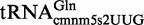, 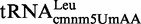, 
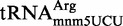
 and 
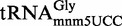
. MnmE is a three-domain protein that binds GTP and methylenetetrahydrofolate (CH_2_-THF), whereas MnmG is a FAD- and NADH-binding protein. The MnmC(o) activity of the bi-functional enzyme MnmC transforms cmnm^5^(s^2^)U to nm^5^(s^2^)U and the MnmC(m) activity of MnmC transforms nm^5^(s^2^)U to the final product mnm^5^(s^2^)U. Thiolation at position 2 of the wobble uridine (U34) in 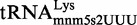, 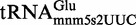 and 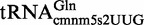 is mediated by the protein MnmA. Modifications occur at the 5- and 2-positions independent of each other. TrmL methylates the 2′-OH group of the U-ribose in 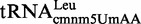 (inset panel).

Proteins MnmE and MnmG (formerly TrmE and GidA, respectively) are evolutionary conserved from bacteria to eukaryotic organelles. In *Escherichia coli*, they are involved in the modification of the wobble uridine of 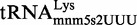, 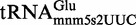, 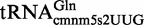, 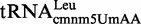, 
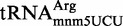
 and 
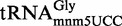
, all of which read NNA/G codons of the split codon boxes, with the exception of 
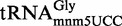
, which reads codons of the glycine family box (11). MnmE and MnmG are dimeric and form a functional α2β2 heterotetrameric complex (MnmEG) in which both proteins are interdependent (12,13). MnmE is a GTP- and tetrahydrofolate (THF)-binding protein, whereas MnmG is a FAD- and NADH-binding protein (14–18). The MnmEG complex catalyzes the addition of the aminomethyl (nm) and carboxymethylaminomethyl (cmnm) groups to position 5 of the wobble uridine using ammonium and glycine, respectively [Figure 1; (13)]. Both reactions require GTP and FAD as well as NADH if FAD is limiting in the *in vitro* reaction. Moreover, a THF derivative, likely methylene-THF, serves as the donor of the methylene carbon that is directly bonded to the C5 atom of U34. However, the detailed mechanism of the basic reaction has not yet been demonstrated (11,13). According to current models, GTP hydrolysis by the MnmE G-domain, which is located distant from the MnmEG active center, leads to structural rearrangements in the MnmEG complex, which are essential for tRNA modification (11,19).

The wobble uridine (U34) of tRNAs modified by MnmEG may be further modified by other enzymes at position 5 or other positions. In 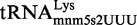, 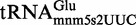 and 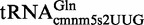, thiolation at position 2 of the U34 is performed by MnmA (20), whereas in 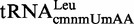, TrmL methylates the 2′-OH group of the U-ribose (21). Therefore, some tRNA substrates for the MnmEG complex are also substrates for MnmA or TrmL.

Moreover, in 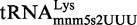, 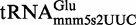, 
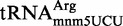
, and 
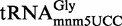
, the resulting products of MnmEG activity are not the final modifications because these tRNAs carry the methylaminomethyl (mnm) group at position 5 of U34. Formation of the mnm^5^-final group is mediated by the action of the two-domain, bi-functional enzyme MnmC (22–26). The oxidoreductase activity of the C-terminal domain, here designated as MnmC(o), is responsible for the FAD-dependent deacetylation that transforms cmnm^5^U into nm^5^U, whereas the methyltransferase activity of the N-terminal domain, designated as MnmC(m), catalyzes the SAM-dependent methylation that transforms nm^5^U to mnm^5^U (Figure 1).

The crystal structure of MnmC consists of two globular domains interacting with each other (27). The structure also reveals that the two catalytic centers of MnmC(o) and MnmC(m) face opposite sides of the protein, thus favoring a model in which the two domains could function in a relatively independent manner. In fact, when two MnmC mutant proteins possessing a catalytically dead MnmC(o) or MnmC(m) domain were mixed, partial recovery of mnm^5^-group synthesis was observed (25). However, considering the relatively hydrophilic nature of the domain interface, the possibility that conformational changes within the entire MnmC protein may occur *in vivo* and affect its functioning, cannot be excluded. A previous attempt to separately express both MnmC domains revealed that MnmC(m) was soluble, whereas MnmC(o) produced inclusion bodies, suggesting that MnmC(m) is required for the correct folding or structural stability of MnmC(o) (25).

How the activities of the MnmEG and MnmC enzymes are organized and modulated remains unclear. Whether tRNAs preferentially utilize one of the two MnmEG pathways, the glycine or the ammonium pathway (Figure 1), *in vivo* depending on metabolic circumstances has not been investigated. The fact that only cmnm^5^, but not nm^5^ or mnm^5^ has been detected to date at position 5 of U34 in 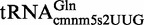 and 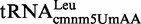 suggests that both tRNAs do not use the ammonium pathway and are not substrates for MnmC(o). Accumulation of the nm^5^s^2^U nucleoside which may have a dual origin [Figure 1; (13)], has not been observed in total tRNA purified from wild-type *E. coli* cells (23). Therefore, the mnm^5^s^2^U synthesis in 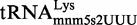 and 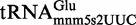 appears to be organized to prevent nm^5^s^2^U accumulation. However, the presence or absence of cmnm^5^s^2^U as an intermediate in mnm^5^s^2^U biosynthesis is difficult to determine from nucleoside analysis of bulk tRNA due to the natural occurrence of cmnm^5^s^2^U in 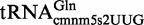. Biosynthetic tuning of mnm^5^s^2^U to avoid the accumulation of intermediates could be achieved by either kinetic tuning of the activities of MnmEG and MnmC, which requires that the sequential modifications are performed at similar or increasing rates or selective degradation of partially modified tRNAs. A previous steady-state kinetic analysis of the activities of the full MnmC protein indicated that the MnmC(m)-dependent reaction (nm^5^→mnm^5^) occurs faster than the MnmC(o)-dependent reaction (cmnm^5^→nm^5^) or at a similar rate at very high substrate (tRNA) concentrations (26). However, this study did not take into account the efficiency of the reactions performed by MnmEG (Figure 1), which is crucial to understand the organization of the mnm^5^s^2^U biosynthetic process.

Modifications located within or adjacent to the anticodon are important for stabilization of codon–anticodon pairing as well as for restricting the dynamics of the anticodon domain and shaping its architecture (1,4). Considering the network of pathways leading to mnm^5^s^2^U production (Figure 1), it is important to determine whether mutations affecting the different activities of MnmC [MnmC(o) or MnmC(m)] have distinct biological consequences and to what extent an accumulation of modification intermediates affects bacterial biology.

In this study, we investigated the activities and the tRNA specificity of the MnmEG and MnmC enzymes *in vivo* and *in vitro*, analyzed how the U34 modification status is influenced by genetic and physiological conditions in bulk and specific tRNAs and assessed the effects of *mnmC* mutations on bacterial growth. Our study was facilitated by the cloning and separate expression of the two MnmC domains, MnmC(o) and MnmC(m). In contrast to a previous report (25), we demonstrate that MnmC(o) can fold independently of MnmC(m) and that the separate domains exhibit similar kinetic properties to those of the full protein. We also show that 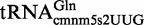 and 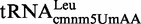 are substrates for MnmC(m), but not for MnmC(o). Our data suggest that MnmEG and MnmC are kinetically tuned to produce only the fully modified nucleoside mnm^5^U in 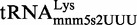. We demonstrate that all the tRNA substrates of MnmEG are modified *in vitro* through the ammonium pathway. However, the net output of the ammonium and glycine pathways of MnmEG *in vivo* depends on growth conditions and tRNA species. Finally, we demonstrate that the loss of any MnmC activity has a biological cost under specific conditions.

## MATERIALS AND METHODS

### Bacterial strains, plasmids, primers, media and general techniques

*Escherichia coli* strains and plasmids are shown in Table 1. A list of the oligonucleotides and primers used in this work is provided in Supplementary Table S1. Transduction with phage P1 was performed as previously described (28). Deletion of the *mnmC(o)* coding region was performed by targeted homologous recombination (29) using the primers MnmC(o)Δ-F and MnmC(o)Δ-R. The MnmC(o)Δ-F primer introduced a TAA stop codon at the end of the MnmC(m) coding region, which is located in the 5′-terminal region of the *mnmC* gene. Deletion of the *mnmC(o)* coding sequence was confirmed by PCR and DNA sequencing. The resulting strain was named IC6629. DNA sequences coding for the MnmC(o) (250–668 a.a.) and MnmC(m) domains (1–250 a.a.) fused to the C-terminal end of a Flag-epitope were amplified by polymerase chain reaction (PCR) from genomic DNA of *E. coli* MG1655 using the specific primer pairs Flag-MnmC(o)F/Flag-MnmC(o)R and Flag-MnmC(m)F/Flag-MnmC(m)R, respectively. The amplicons were cloned into pBAD TOPO TA for expression under control of the AraC-P_BAD_ system. The resulting plasmids were transformed into a *mnmC::kan* strain, unless otherwise specified. A DNA fragment encoding the MnmC(m) domain fused at its N-terminal end to a 6x-His-epitope was cloned into the same vector after PCR amplification with the His-MnmC(m)R/His-MnmC(m)F primer pair. LBT (LB broth containing 40 mg/l thymine) and LAT (LBT containing 20 g of Difco agar per liter) were used for routine cultures and plating of *E. coli*, respectively, unless otherwise specified. When required, antibiotics were added at the following final concentrations: 100 µg/ml ampicillin; 12.5 µg/ml tetracycline; 25 µg/ml chloramphenicol and 80 µg/ml kanamycin. Cell growth was monitored by measuring the optical density of the cultures at 600 nm (OD_600_).

**Table 1.** Strains and plasmids used in this study

Strain or plasmid	Description	References
***Eschirichia coli* strains**		
DEV16 (IC4385)^a^	*thi-1 rel-1 spoT1 lacZ105_UAG_ val*^R^*mnmE*-Q192X	(14,48)
BW25113 (IC5136)^a^	*lacI*^q^ *rrnB_T14_* Δ*lacZ*_WJ16_ *hsdR514* Δ*araBAD*_AH33_ Δ*rhaBAD*_LD78_	(29)
TH178 (IC5255)^a^	*mnmA1 fadR::*Tn*10*	(45)
MG1655(IC5356)^a^	F^-^	D. Touati
TH48 (IC6017)^a^	ara, Δ(lac-proB), nalA, argE_am_, rif, thi, *fadL::*Tn*10*	(23)
TH49 (IC6018)^a^	TH48 *mnmC(m)*-G68D	(23)
TH69 (IC6019)^a^	TH48 *mnmC-*W131stop	(23)
IC4639	DEV16 mnmE^+^ *bgl* (Sal^+^)	(12)
IC5241	MG1655 *mnmG::*Tn*10* [Tet^R^]	(12)
IC5358	MG1655 *mnmE::kan*	(43)
IC5397	P1 (IC5255) x IC4639; [IC4639 *mnmA-*Q233stop]	M. Villarroya
IC5827	BW25113 *mnmE::kan*	NBRP- Japan
IC5854	BW25113 *trmL::kan*	(21)
IC5937	IC4639 *mnmA::kan* [or IC4639 *ΔmnmA*]	(39)
IC5975	BL21-DE3 *mnmG::kan*	(17)
IC6010	BW25113 *mnmC::Kan* [or BW25113 *ΔmnmC*]	(13)
IC6166	IC5975 carrying pIC1446	(17)
IC6222	P1 (IC6010) x IC4639; [IC4639 *ΔmnmC*)]	This study
IC6374	IC4639 *trmL::kan* [IC4639 *ΔtrmL*]	(21)
IC6411	P1 (IC5241) x IC6374 (*trmL::kan*) [IC4639 *ΔtrmL ΔmnmG*]	(21)
IC6424	IC5358 carrying pIC684	(14)
IC6587	P1 (TH49) x IC4639; [IC4639 *mnmC(m)*-G68D]	This study
IC6588	P1 (TH49) x IC5937; [IC4639 *ΔmnmA mnmC(m)*-G68D*]*	This study
IC6589	P1 (IC6010) x IC5397; [IC4639 *mnmA*-Q233stop *ΔmnmC*]	This study
IC6629	BW25113 *mnmC(o)::cat* [or BW25113 *ΔmnmC(o)*]	This study
IC6725	P1 (IC6629) x IC5937 [IC4639 *ΔmnmA ΔmnmC(o)*]	This study
**Plasmids**		
pIC684	GST fusion of *mnmE* cloned in pGEX-2T	(14)
pIC1083	*Eschirichia coli* 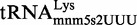 cloned in pUC19	(34)
pIC1253	*flag-mnmC* cloned in pBAD-TOPO	(13)
pIC1339	*flag-mnmC(o)* cloned in pBAD-TOPO	This study
pIC1340	*flag-mnmC(m)* cloned in pBAD-TOPO	This study
pIC1394	*Eschirichia coli* 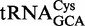 cloned into pUC19, SmaI site	This study
pIC1395	*E schirichia coli* 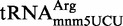 cloned into pUC19, SmaI site	This study
pIC1446	*his-mnmG* cloned in pET15b	(17)
pIC1535	*Eschirichia coli* 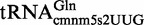 cloned into pBAD-TOPO	This study
pIC1536	*Eschirichia coli* 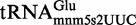 cloned into pUC19, SmaI site	This study
pIC1537	*Eschirichia coli* 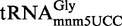 cloned into pUC19, SmaI site	This study
pIC1550	*Eschirichia coli* 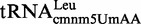 cloned into pUC19, SmaI site	This study
pIC1577	pBSKrna	(32)
pIC1617	*Eschirichia coli* 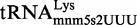 cloned in pBSKrna, EcoRV site	This study
pIC1664	*Eschirichia coli* 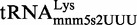 cloned in pBSKrna, EcoRI/PstI sites	This study
pIC1665	*Eschirichia coli* 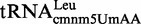 cloned into pBSKrna, EcoRI–PstI site	This study
pIC1677	*his-mnmC(m)* cloned in pBAD-TOPO	This study
pIC1714	*Eschirichia coli* 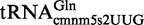 cloned into pBSKrna, EcoRI–PstI site	This study

^a^Name in our collection between brackets.

### Growth rate determinations and competition experiments

To determine bacterial growth rates, overnight cultures were diluted 1/100 in fresh LBT medium and incubated at 37°C with shaking. The doubling time was measured by monitoring the optical density of the culture at 600 nm. Samples were taken from exponentially growing cultures after at least 10 generations of steady-state growth. The growth rate was calculated as the doubling time of each culture in the steady-state log phase by linear regression. Competition experiments were performed as previously reported (21). Briefly, the reference strain (BW25113) and the strain to be tested (TH48, TH49 or TH69) were grown separately to stationary phase by incubation at 37°C. Equal volumes of both strains were mixed and a sample was immediately taken to count viable cells on LAT plates with and without the antibiotic required to estimate the content of each strain (tetracycline to identify strains of the TH48 background). Six cycles of 24-h growth at 37°C (with shaking) were performed by diluting mixed cultures of 1/1000 in LBT medium. After the sixth cycle, the mixed culture was analyzed for its strain content as before. The final ratio was calculated as the ratio of the number of colony forming units (CFU) per milliliter recovered on LAT supplemented with tetracycline versus CFU per milliliter on LAT.

### Purification of recombinant proteins

Routinely, a single colony was inoculated into 5 ml LBT supplemented with adequate antibiotics and grown overnight at 37°C. The pre-culture was then inoculated (1:100 dilution) into 1000 ml LBT plus antibiotic and grown until the OD_600_ reached ∼0.5. Protein expression was induced by the addition of 0.2% arabinose (Flag-tagged proteins). The cultures were grown for an additional 3–4 h at 30°C with gentle shaking, harvested by centrifugation (4500*g* for 15 min at 4°C), washed with TBS buffer (50 mM Tris–HCl, pH 7.4, 150 mM NaCl and 5 mM MgCl_2_) and stored at −20°C. For enzyme purification, frozen cells were thawed on ice, resuspended in 10 ml of lysis buffer (TBS containing 1 mM PMSF and 1 mM EDTA) and disrupted by sonication. The lysate was centrifuged at 10 000*g* for 45 min at 4°C and the supernatant was mixed with 0.3–0.6 ml of pre-equilibrated anti-Flag agarose resin (ANTI-FLAG M2 Affinity Gel, A2220-Sigma) and incubated at 4°C for 1 h with mild shaking. Instructions from the manufacturer were followed for washing and eluting Flag-tagged proteins. The fractions containing the proteins were pooled, concentrated with Amicon Ultra-15 30k devices in TBS buffer and stored in 50-µl fractions with 15% glycerol at −20°C. The His-MnmC(m) domain was purified using Clontech’s TALON Metal Affinity Resin according to the manufacturer’s instructions. The MnmE and MnmG proteins were purified as previously described (14,17). Protein concentrations were determined with a NanoDrop spectrophotometer at 280 nm. The purity of all enzymes was >95% as estimated by sodium dodecyl sulphate–polyacrylamide gel electrophoresis (SDS–PAGE) and coomassie blue staining.

### FAD cofactor analysis

FAD was released from recombinant proteins by heating at 75°C for 5 min in the dark and analyzed by high-performance liquid chromatography (HPLC). Briefly, HPLC separation was achieved with a Synergi 4u Fusion column (25 cm × 4.6 mm, 5 µm) using a gradient of 5 mM ammonium acetate, pH 6.5, to acetonitrile 50% and water 50% over 35 min at a flow rate of 0.6 ml/min. Flavins were detected by fluorescence emission (525 nm) using a Waters 474 scanning fluorescence detector set at 450 nm for excitation.

### Stability of MnmC recombinant proteins

Strain IC6010 carrying pIC1253, pIC1339 or pIC1340 was grown overnight in LBT with ampicillin at 37°C. Overnight cultures were diluted 1/100 in the same medium supplemented with 0.1% L-arabinose (inducer of the AraC-P_BAD_ system) and incubated at 37°C for 2 h. To halt the expression of the MnmC recombinant proteins, the cells were recovered by centrifugation at 3000*g* for 10 min, washed once with fresh LBT medium, resuspended in the same volume of pre-warmed LBT containing ampicillin and 1% glucose (repressor of the AraC-P_BAD_ system) and incubated at 37°C. Samples were taken several times after the addition of glucose. The cells were lysed by brief ultrasonication and the lysate was centrifuged at 10 000*g* for 10 min at 4°C. The soluble fractions (50–150 μg of total proteins) were analyzed by western blotting using anti-Flag and anti-GroEL antibodies.

### Gel filtration analysis of protein interactions

To investigate whether Flag-MnmC(o) interacts with Flag-MnmC(m), each protein was mixed at a final concentration of 5 µM in a final volume of 100 µl in TBS buffer containing 5 mM DTT and 3% glycerol and incubated at room temperature for 2 h. The same procedure was used to study the interaction of the full MnmC protein with MnmE or MnmG. The samples were analyzed by gel filtration using a Superdex 75 HR (MnmC domains) or a Superdex 200 HR (MnmC/MnmE/MnmG) column at a flow rate of 0.7 or 0.3 ml/min, respectively, in TBS Buffer containing 5 mM DTT. Gel filtration markers were used to calibrate the columns. The proteins were detected by ultraviolet (UV) absorbance at 280 nm.

### Surface plasmon resonance evaluation of the MnmC(o)–MnmC(m) interaction

Surface plasmon resonance (SPR)-based kinetic analysis was used to determine the affinity between the recombinant MnmC(o) and MnmC(m) proteins. An anti-His monoclonal antibody (Roche, 100 ng/μl in 10 mM sodium acetate, pH 4.5) was immobilized onto a CM-5 sensor chip (Biacore AB, Uppsala, Sweden) at 7000 resonance units (RUs) using an amine coupling kit (Biacore AB) according to the manufacturer’s instructions. His-MnmC(m) at 2 μM in TBS buffer containing 0.005% Tween-20 surfactant was immobilized by capturing (∼600 RUs). Subsequently, various concentrations of Flag-MnmC(o) protein in TBS buffer were passed over the sensor chip at a flow rate of 30 μl/min at 25°C and the interactions were monitored for 1 min. The sensor surface was washed with TBS buffer to detect dissociation and then regenerated with a pulse of 5 mM NaOH (10 s at 60 μl/min). The data were evaluated with BiaEvaluation 3.1 Software (Biacore AB, Uppsala, Sweden).

### Isolation of bulk tRNA from *E. coli* and reverse-phase HPLC analysis of nucleosides

Total tRNA purification and analysis of nucleosides by reverse-phase HPLC were performed as described previously (13,35,36). HPLC analysis was monitored at appropriate wavelengths to achieve optimal adsorption of the target nucleosides, 314 nm for thiolated nucleosides and 254 nm for non-thiolated nucleosides. The nucleosides were identified according to their UV spectra (35) and by comparison with appropriate controls.

### Isolation of specific chimeric and native tRNAs

The *E. coli* 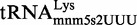, 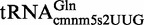 and 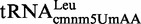 genes were cloned into pBSKrna (37) digested with either EcoRV (to produce chimeric tRNAs in which the *E. coli* tRNA is inserted into a human cytosolic 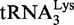 sequence that is used as a scaffold) or EcoRI and PstI to produce a tRNA without the scaffold (overexpressed ‘native’ tRNAs). The overproduction of tRNAs in strains transformed with pBSKrna derivative plasmids was performed as described previously (37). Specific chimeric tRNAs were purified from bulk tRNA by the Chaplet Column Chromatography method (38) using a biotinylated DNA probe that is complementary to the scaffold human cytosolic 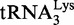 moiety (common to all chimeric tRNAs). The probe was immobilized on a HiTrap Streptavidin HP column. The same approach was used to purify overexpressed ‘native’ and true native tRNAs using biotinylated DNA probes that are complementary to the specific sequence of each *E. coli* tRNA (Supplementary Table S1).

### *In vitro* transcription of *E. coli* tRNAs

The *E. coli* genes encoding 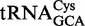, 
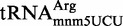
, 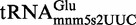, 
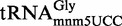
 and 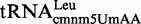 were PCR-amplified from genomic DNA using ‘vent’ polymerase and the primers Cys-F/Cys-R, Arg-F/Arg-R, Glu-F/Glu-R, Gly-F/Gly-R and Leu-F/Leu-R, respectively. The amplicons were cloned into a SmaI-linearized pUC19 plasmid to produce pIC1394, pIC1395, pIC1536, pIC1550 and pIC1537. The *E. coli* gene encoding 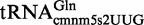 was PCR-amplified from genomic DNA using expand-long polymerase and the primers Gln-F and Gln-R and cloned into pBAD-TOPO. The plasmid pIC1083 (containing the 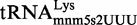 gene), a derivative of pUC19, was a gift from Dr Tamura (34). Unmodified *E. coli* tRNAs were prepared by *in vitro* transcription from BstNI-digested plasmids (pIC1083, pIC1394 and pIC1395) and HindIII-digested plasmids (pIC1535, pIC1536, pIC1550 and pIC1537) using the Riboprobe T7 transcription kit (PROMEGA) and 2–5 μg of each digested plasmid as a DNA template in a 50-μl reaction mix.

### *In vitro* and *in vivo* activity of the recombinant MnmC(o) and MnmC(m) proteins

To analyze the *in vitro* activity of the recombinant proteins, total tRNA (40 µg) from a *mnmC-*W131stop or *mnmC(m)-*G68D mutant was incubated in a 200-μl reaction mixture containing 50 mM Tris–Cl (pH 8.0), 50 mM ammonium acetate, 0.5 mM FAD or 0.5 mM S-Adenosyl-L-Methionine (SAM), 5% glycerol and 2 μM of the purified protein [Flag-MnmC, Flag-MnmC(o), or Flag-MnmC(m)] for 40 min at 37°C. tRNA was recovered by phenol extraction and ethanol precipitation and subsequently treated with nuclease P1 and *E. coli* alkaline phosphatase. The resulting nucleosides were analyzed by reverse-phase HPLC as described (13,30). For *in vivo* complementation studies, overnight cultures of strains *mnmC-*W131stop or *mnmC(m)-*G68D containing pBAD-TOPO or derivative plasmids (pIC1340 and pIC1339) were diluted 1:100 in 100 ml LBT containing 0.2% arabinose and grown to an OD_600_ of 0.5. The cells were recovered by centrifugation at 4500*g* for 15 min at 4°C. Bulk tRNA was obtained and analyzed by HPLC as described above.

### Kinetic analysis of MnmC- and MnmEG-catalyzed modifications

To assay MnmC(o) or MnmC(m) activity, the reaction mixture (100 μl) contained 50 mM Tris–HCl (pH 8), 50 mM ammonium acetate, 3% glycerol, 2 mM NaCl, 73 µM MgCl_2_, tRNA (0.5–5 μM), Flag-tagged protein (25 nM) and 100 μM FAD or SAM (depending on the MnmC activity to be determined). The mixtures were pre-incubated at 37°C for 3 min before addition of proteins. All reactions were performed at 37°C. The reactions were halted after 60–90 s of incubation by adding 100 μl of 0.3 M sodium acetate, pH 5.2. To assay the ammonium-dependent MnmEG activity with respect to tRNA concentration, the reaction mixture (100 μl) contained 100 mM Tris–HCl, pH 8, 100 mM ammonium acetate, 5 mM MgCl_2_, 5% glycerol, 5 mM DTT, 0.5 mM FAD, 2 mM GTP, 1 mM methylene-THF, 10 µg bovine serum albumin (BSA) and tRNA (0.1–2 μM). The reaction was initiated by the addition of 0.1 µM MnmE•MnmG complex obtained as previously described (13) and stopped after 2 min of incubation at 37°C by adding 100 μl 0.3 M sodium acetate, pH 5.2. In all cases, tRNA was finally recovered by phenol extraction and ethanol precipitation and treated with nuclease P1 and *E. coli* alkaline phosphatase. The resulting nucleosides were analyzed by reverse-phase HPLC. The area of the synthesized nucleoside was calculated and its amount was extrapolated from standard curves that were prepared using chemically synthesized nucleosides (A. Malkiewicz, University of Lodz, Poland) over a range of 0–250 ng. Experiments were performed in triplicate. To determine the *V*_max_ and *K_m_* values, the data were fitted to the Michaelis–Menten equation using nonlinear regression (GraphPad Prism v4.0).

### Analysis of the tRNA substrate specificity of MnmC(o) and MnmC(m) *in vitro*

To analyze the specificity of MnmC(o) and MnmC(m) *in vitro*, the tRNA (10–15 μg) was first modified using the MnmEG complex through the ammonium and glycine pathways as described previously (13). The modified tRNA was phenolized, ethanol precipitated and incubated with 2 μM MnmC(o) or MnmC(m) domains in 200 μl (total volume) of MnmC buffer containing 50 mM Tris–HCl (pH 8.0), 50 mM ammonium acetate, 0.5 mM FAD [for the MnmC(o) assay] or 0.5 mM SAM (for the MnmC(m) assay) and 5% glycerol. After incubating for 40 min at 37°C, the tRNA was recovered by phenolization and ethanol precipitation and the tRNA was then treated with nuclease P1 and *E. coli* alkaline phosphatase. The resulting nucleosides were analyzed by reverse-phase HPLC as described above.

### Acid resistance assay

The acid resistance experiments were performed essentially as previously reported (39). Briefly, strains were grown in LBT containing 0.4% glucose to stationary phase. The cultures were then diluted 1:1000 into EG medium [minimal E medium containing 0.4% glucose; (40)], pH 2.0, supplemented or not with 0.7 mM glutamate. Samples were obtained at 0, 1, 2, 3 and 4 h post acid challenge and spotted on LAT plates.

## RESULTS

### General roles of the MnmEG and MnmC enzymes in the modification status of tRNAs

In a previous study, we demonstrated that MnmEG catalyzes two different reactions *in vitro* and produces nm^5^ and cmnm^5^ using ammonium and glycine, respectively, as substrates (13). We also reported the presence of cmnm^5^s^2^U and nm^5^s^2^U in total tRNA purified from an *mnmC* null mutant. However, it has been suggested that the formation of these intermediates is sensitive to growth conditions and specific strains, which might explain why they were not detected in other studies (23,26,41).

To obtain a clear picture of the functional activity of enzymes MnmEG and MnmC during exponential growth (mid-log phase; OD_600_ of ∼0.6) in LBT, we analyzed the HPLC profile of bulk tRNA isolated from several strains and different genetic backgrounds (Table 2 and Supplementary Figure S1). In the HPLC analysis, absorbance was monitored at 314 nm to maximize the detection of thiolated nucleosides. We consistently detected a small amount (∼10–20%) of cmnm^5^s^2^U in the wild-type strains of three different backgrounds, TH48, BW25113 and MG1655 (Table 2), which may originate from 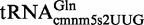 or might be an intermediate of the final product mnm^5^s^2^U that is present in 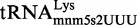 and 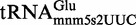. Notwithstanding, mnm^5^s^2^U was the major final product (∼80–90%). We were unable to identify nucleosides mnm^5^U and cmnm^5^Um (which are present in 
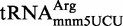
, 
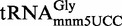
 and 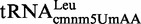) when tRNA hydrolysates were monitored at 254 nm, as a comparison of chromatograms of total tRNA purified from wild-type strains and their *mnmE* or *mnmG* derivatives did not allow the detection of any peaks attributable to those nucleosides in the wild-type chromatogram.

**Table 2.** Relative distribution of nucleosides in bulk tRNA purified from exponentially growing strains

Strain	Relative distribution (%) of nucleosides^a^			
	nm^5^s^2^U	mnm^5^s^2^U	cmnm^5^s^2^U	s^2^U
**TH48 background**				
wt		83 ± 5	17 ± 5	
*mnmC-*W131stop	31 ± 1		69 ± 1	
*mnmC(m)*-G68D	85 ± 5		15 ± 5	
**BW25113 background**				
wt		83 ± 3	17 ± 3	
*ΔmnmG or ΔmnmE*				100
*ΔmnmC*	27 ± 4		73 ± 4	
*ΔmnmC(o)*		14 ± 3	86 ± 3	
**MG1655 background**				
wt		85 ± 3	15 ± 3	
*ΔmnmG*				100

^a^tRNA was purified and degraded to nucleosides for HPLC analysis. The percentage of nucleosides represents the distribution of the peak area of each nucleoside compared to the sum of the peak areas of the two nucleosides considered. Each value represents the mean of at least two independent experiments. wt: wild-type.

As expected, the intermediates cmnm^5^s^2^U and nm^5^s^2^U were observed in *mnmC* null mutants (carrying *mnmC*-W131stop or *ΔmnmC* mutations); these nucleosides were typically distributed in a 70/30 ratio, suggesting that the MnmEG enzyme preferably uses the glycine pathway under the growth conditions used in these experiments (Table 2). This conclusion was consistent with the cmnm^5^s^2^U/mnm^5^s^2^U ratio observed in the *ΔmnmC(o)* mutant (∼86/14), which clearly exhibited a MnmC(o)^−^ MnmC(m)^+^ phenotype. Moreover, the relative distribution of cmnm^5^s^2^U and nm^5^s^2^U in the *mnmC(m)*-G68D mutant was ∼15/85, suggesting that most of the cmnm^5^s^2^U formed by the glycine pathway is converted to nm^5^s^2^U by the MnmC(o) activity present in this strain and that the remaining cmnm^5^s^2^U could proceed, at least partially, from 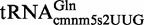. Taken together, these results clearly support the idea that MnmEG uses ammonium or glycine to modify tRNAs *in vivo* and that MnmC(m) functions independently of MnmC(o) [see strain *ΔmnmC(o)* in Table 2] to transform tRNAs modified by MnmEG via the ammonium pathway (Figure 1), which raises the possibility that certain tRNAs may be substrates for MnmC(m), but not for MnmC(o).

### Expression and purification of the MnmC(o) and MnmC(m) domains

**Figure 2.**
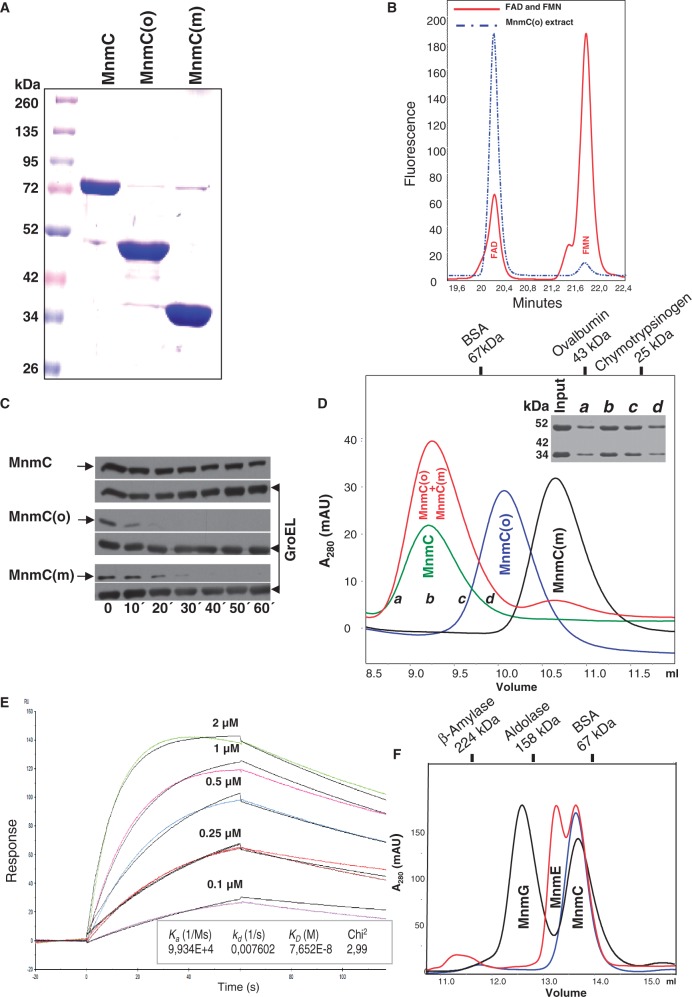
Characterization of the MnmC recombinant proteins. (**A**) Coomassie blue staining of SDS–PAGE containing the purified Flag-MnmC, Flag-MnmC(o) and Flag-MnmC(m) proteins used in this work. (**B**) HPLC analysis of the Flag-MnmC(o) extract (blue line) and markers (red line). FMN: flavin mononucleotide. (**C**) Half-life of the Flag-MnmC proteins over time as determined by tracking their decline after the addition of glucose to cultures of IC6010 (*ΔmnmC*) transformed with the plasmids pIC1253, pIC1339 or pIC1340, which express Flag-MnmC, Flag-MnmC(o) and Flag-MnmC(m), respectively. GroEL was used as a loading control. Protein levels were detected by western blotting. (**D**) Gel filtration analysis of the purified Flag-MnmC proteins [full MnmC protein: green; MnmC(o): blue and MnmC(m): black] and a Flag-MnmC(o)/Flag-MnmC(m) mix (red). The elution positions of the size markers are indicated on the top. Elution fractions *a–d* from the chromatography of the Flag-MnmC(o)/Flag-MnmC(m) mix were pooled for further analysis. Inset: SDS–PAGE of elution fractions *a–d* from the Flag-MnmC(o)/Flag-MnmC(m) mix. Fractions (500 μl) were precipitated with trichloroacetic acid before loading. The gel was stained with coomassie blue. The marker molecular masses are indicated on the left. (**E**) SPR analysis of the MnmC(o)–MnmC(m) interaction. Flag-MnmC(o) was injected into a solution passing over a sensor chip containing the immobilized His-MnmC(m) (600 RU). Representative sensorgrams for various concentrations of Flag-MnmC(o) are shown. (**F**) Gel filtration analysis of the purified MnmC protein (blue line) and a mix of MnmC with MnmG (black line) or MnmE (red line). The elution positions of the size markers are indicated on the top. Proteins were detected by UV absorbance at 280 nm.

In order to study the functional independence of the two MnmC domains, we decided to express them separately. The full *mnmC* gene and its two domains, *mnmC*(*o*) (encoding for amino acids 250–668 of MnmC) and *mnmC*(*m*) (encoding for amino acids 1–250 of MnmC)*,* were N-terminal Flag-tagged by PCR and cloned into the pBAD-TOPO expression vector. Recombinant protein expression was induced in the *E. coli* mutant *mnmC::kan* (*ΔmnmC*) by adding 0.2% arabinose. The Flag-tagged proteins MnmC, MnmC(o) and MnmC(m) were purified close to homogeneity and exhibited apparent molecular masses of 78, 48 and 34 kDa, respectively (Figure 2A).

The Flag-MnmC protein and its C-terminal domain [Flag-MnmC(o)] were yellow, and their spectra showed maximum absorption peaks at ∼375 and 450 nm, indicating the presence of a flavin derivative. The putative cofactor was identified as FAD by its retention time (Figure 2B). Therefore, the isolated MnmC(o) domain contains non-covalently bound FAD, which suggests that it is correctly folded. To our knowledge, this is the first report describing the expression and purification of the soluble form of MnmC(o). A previous attempt to purify this domain was unsuccessful because overexpression of a somewhat different, recombinant MnmC(o) produced inclusion bodies, which led to the hypothesis that MnmC(o) is incapable of folding on its own and requires the presence of MnmC(m) (25). Our data, however, demonstrate that MnmC(o) can fold independently of MnmC(m). Interestingly, the *in vivo* stability of the separated domains was significantly lower than that of the full protein (Figure 2C), suggesting that the physical interaction of both domains in the full protein confers greater stability.

The full MnmC protein and the separate domains MnmC(o) and MnmC(m) behaved as monomeric proteins when subjected to gel filtration chromatography (Figure 2D). However, a mixture of MnmC(o) and MnmC(m) displayed the same elution profile as the full protein, indicating that the separate domains interact *in vitro* (Figure 2D). This interaction was further explored by kinetic analysis using surface plasmon resonance (SPR). Flag-MnmC(o) was injected at different concentrations onto the immobilized His-MnmC(m) ligand (Figure 2E). The apparent equilibrium constant *K*_D_ was 87 ± 15 nM, which is indicative of a strong interaction between MnmC(o) and MnmC(m).

Taking into consideration the functional relationships of MnmC with the MnmEG complex, we explored whether MnmC interacts with members of the complex, i.e. MnmE or MnmG. A final concentration of 5 µM Flag-MnmC was mixed with 5 µM His-MnmG or MnmE and the samples were analyzed by gel filtration. As shown in Figure 2F, no interaction between the full MnmC protein and MnmE or MnmG was observed. The same negative result was obtained by SPR (data not shown).

### *In vitro* and *in vivo* determination of the enzymatic activities of MnmC(o) and MnmC(m)

**Figure 3.**
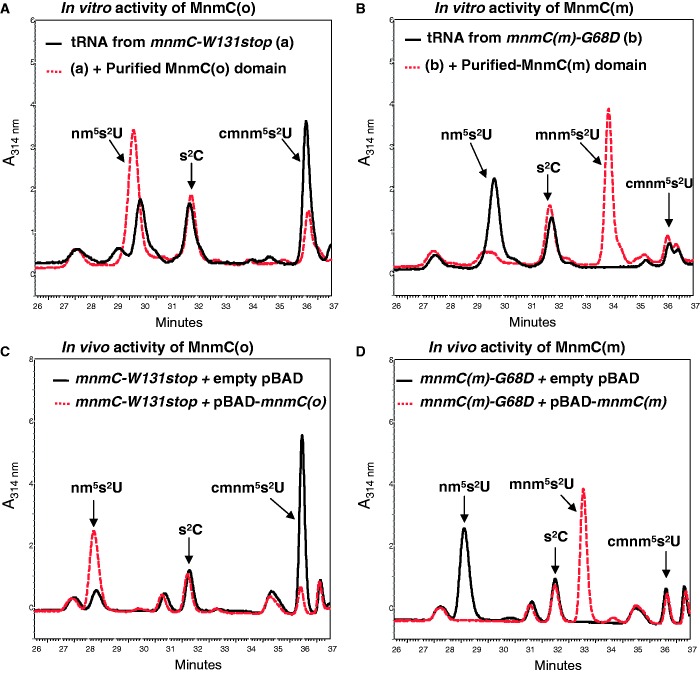
*In vitro* and *in vivo* activity of the recombinant proteins MnmC(o) and MnmC(m). (**A**) and (**B**) HPLC analysis of total tRNA from a null *mnmC* mutant (IC6019; panel A) and an *mnmC(m)*-G68D mutant (IC6018; panel B) before (solid black line) and after *in vitro* incubation (dotted red line) with purified MnmC(o) and MnmC(m) recombinant proteins, respectively. (**C**) and (**D**) HPLC analysis of total tRNA extracted from IC6019 (panel C) and IC6018 (panel D) transformed with pBAD-TOPO (solid black lines) or a pBAD-TOPO derivative expressing Flag-MnmC(o) and Flag-MnmC(m), respectively (dotted red lines). Absorbance was monitored at 314 nm to maximize the detection of thiolated nucleosides. Small variations in elution times between upper and lower panels were probably due to negligible variations in buffers prepared in different days. Note that nucleosides were identified by both elution times and spectra (data not shown).

To analyze whether the recombinant MnmC(o) and MnmC(m) proteins are functionally active, we first assessed their tRNA modifying capability *in vitro* using total tRNA purified from exponentially growing cultures as a substrate. The *in vitro* activity of the recombinant MnmC(o) protein was investigated by incubating tRNA purified from the null mutant *mnmC*-W131stop (nm^5^s^2^U/cmnm^5^s^2^U ratio ≈ 31/69, Table 2) with 2 µM Flag-MnmC(o) domain and 0.5 mM FAD. As shown in Figure 3A, a major part of cmnm^5^s^2^U was converted to nm^5^s^2^U, which demonstrates that Flag-MnmC(o) is catalytically active in the *in vitro* assay, although a small part of cmnm^5^s^2^U, probably proceeding from 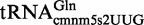, remained unaltered. When tRNA purified from the *mnmC(m)*-G68D mutant (nm^5^s^2^U/cmnm^5^s^2^U ratio ≈ 85/15; Table 2) was incubated with 2 µM Flag-MnmC(m) and 0.5 mM SAM, nm^5^s^2^U was modified to mnm^5^s^2^U, demonstrating the capability of the recombinant protein to perform the methylation reaction *in vitro* (Figure 3B). As expected, the small peak corresponding to cmnm^5^s^2^U (according to its retention time and spectrum) remained unchanged after the *in vitro* reaction mediated by MnmC(m) (Figure 3B).

Then, we assessed the capability of the recombinant MnmC(o) and MnmC(m) proteins to modify tRNA *in vivo*. The strain with the null mutation *mnmC*-W131stop (IC6019) was transformed with pBAD-TOPO and its derivative pIC1339 expressing MnmC(o), whereas strain *mnmC(m)*-G68D (IC6018) was transformed with pBAD-TOPO and its derivative pIC1340 expressing MnmC(m). In the resulting strains, which were grown to exponential phase in the presence of the arabinose inducer, the recombinant MnmC(o) protein catalyzed the conversion of cmnm^5^s^2^U into nm^5^s^2^U, although a peak of remnant cmnm^5^s^2^U was observed (Figure 3C) and nm^5^s^2^U was fully transformed to mnm^5^s^2^U by MnmC(m) (Figure 3D). Similar results were obtained in the absence of arabinose (data not shown). This finding indicates that recombinant MnmC(o) and MnmC(m) are expressed and accumulate to some extent in the absence of the inducer, although it was not possible to detect them by western blotting with an anti-Flag antibody (data not shown). The fact that both proteins were capable of modifying tRNA despite their very low concentrations suggests that these proteins have high activity *in vivo*.

### Kinetic analysis of the MnmC- and MnmEG-dependent reactions

To explore whether the recombinant MnmC(o) and MnmC(m) proteins display similar kinetic properties to those exhibited by the entire MnmC protein, we conducted steady-state kinetic experiments. The conditions used for each reaction were similar to enable a comparison of the kinetic constants and were based on conditions known to optimize activity (23,26). The substrate for the assays was a chimeric version of *E. coli* 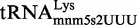 expressed from pBSKrna (37,42), which facilitates overproduction and purification of recombinant RNA and has been successfully used by our group in previous studies (21,33). Here, the 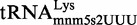 gene was inserted into the EcoRV site of the region encoding the scaffold tRNA (a human cytosolic 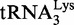 lacking the anticodon region). The resulting chimeric tRNA essentially contained the complete *E. coli* 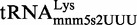 sequence fused at its 5′- and 3′-ends to the truncated anticodon stem of human cytosolic 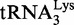, producing an RNA of ∼170 nt (Supplementary Figure S2).

The FAD-dependent-oxidoreductase activity of the recombinant MnmC(o) and MnmC proteins was compared by determining the conversion of cmnm^5^s^2^U to nm^5^s^2^U on the cmnm^5^s^2^U-containing chimeric 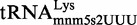 (chi-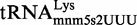) purified from an *mnmC* null mutant (IC6010). The SAM-dependent methyltransferase activity of MnmC(m) and MnmC was compared by following the conversion of nm^5^s^2^U to mnm^5^s^2^U on the nm^5^s^2^U-containing chi-
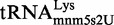
 extracted from an *mnmC(m)*-G68D mutant (IC6018). All proteins displayed Michaelis–Menten kinetics with respect to varying concentrations of chi-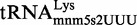 (Supplementary Figure S3). As shown in Table 3, the enzymatic activity of each isolated MnmC domain had kinetic parameters (*k*_cat_ and *K*_m_) similar to those exhibited by the entire protein, which supports the notion that the MnmC domains function independently.

**Table 3.** Kinetic parameters with respect to chi-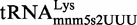 for reactions catalyzed by MnmC, MnmC(o), MnmC(m), and MnmEG

Reaction	*K_m_* (μM)	*V*_max_ (nmoles min^−1 ^mg^−1^)	*k*_cat_ (s^−1^)	*k*_cat_*/K*_m_ (s^−1 ^μM^-1^)
MnmC_(FAD)_ (cmnm^5^s^2^U → nm^5^s^2^U)	15.7 ± 3.4	457 ± 12	0.59 ± 0.02	0.038
MnmC(o) domain_(FAD)_ (cmnm^5^s^2^U → nm^5^s^2^U)	6.1 ± 2.1	486 ± 69	0.39 ± 0.05	0.064
MnmC_(SAM)_ (nm^5^s^2^U → mnm^5^s^2^U)	4.4 ± 1.1	365 ± 77	0.46 ± 0.10	0.105
MnmC(m) domain_(SAM)_ (nm^5^s^2^U → mnm^5^s^2^U)	4.2 ± 1.1	895 ± 179	0.52 ± 0.10	0.124
MnmEG_(NH4)_ (s^2^U → nm^5^s^2^U)	0.6 ± 0.2	5.9 ± 0.3	0.012 ± 0.001	0.020

The values are the mean ± SD of a minimum of three independent experiments.

The *k*_cat_ constants of the MnmC activities were similar to those of previous studies (23,26), although the *K*_m_ values were higher than those reported by Pearson and Carell (26), most likely due to the nature of the tRNA substrate used in our experiments. In any case, our data indicated that MnmC(m) displayed a catalytic efficiency (*k*_cat_/*K*_m_) 2-fold higher than that of MnmC(o) (∼0.1 versus ∼0.05; Table 3), which is in agreement with the proposal that synthesis of the final modification mnm^5^(s^2^)U is favored by kinetic tuning of the MnmC activities, thus avoiding accumulation of the nm^5^(s^2^) intermediate (26).

We also analyzed the catalytic parameters of the ammonium-dependent reaction mediated by the MnmEG complex, whose activity precedes that of MnmC(m) in the mnm^5^(s^2^)U synthesis (Figure 1). We used conditions known to be appropriate for assaying the MnmEG activities *in vitro* (13) and included the presence of Mg^2+^ (5 mM), which is required for GTP hydrolysis by MnmE (43). In line with this, the assay buffer differed from that of the MnmC reactions where a relatively low concentration of Mg^2+^ was used (73 μM), as MnmC activities are known to be severely inhibited at 5 mM of Mg^2+^ (23). Under the proper *in vitro* conditions for each enzyme, the modification reaction mediated by MnmEG (incorporation of nm^5^ into U34) displayed an ∼5-fold lower catalytic efficiency than that of the MnmC(m)-mediated reaction (Table 3). However, this comparison should be handled with caution because the conditions used in the MnmEG and MnmC(m) assays were different, and the *K*_m_ values may change according to the buffer conditions. In addition, the structure of the chimeric tRNA used as a substrate could affect the catalytic cycle of each enzyme in a different manner.

**Figure 4.**
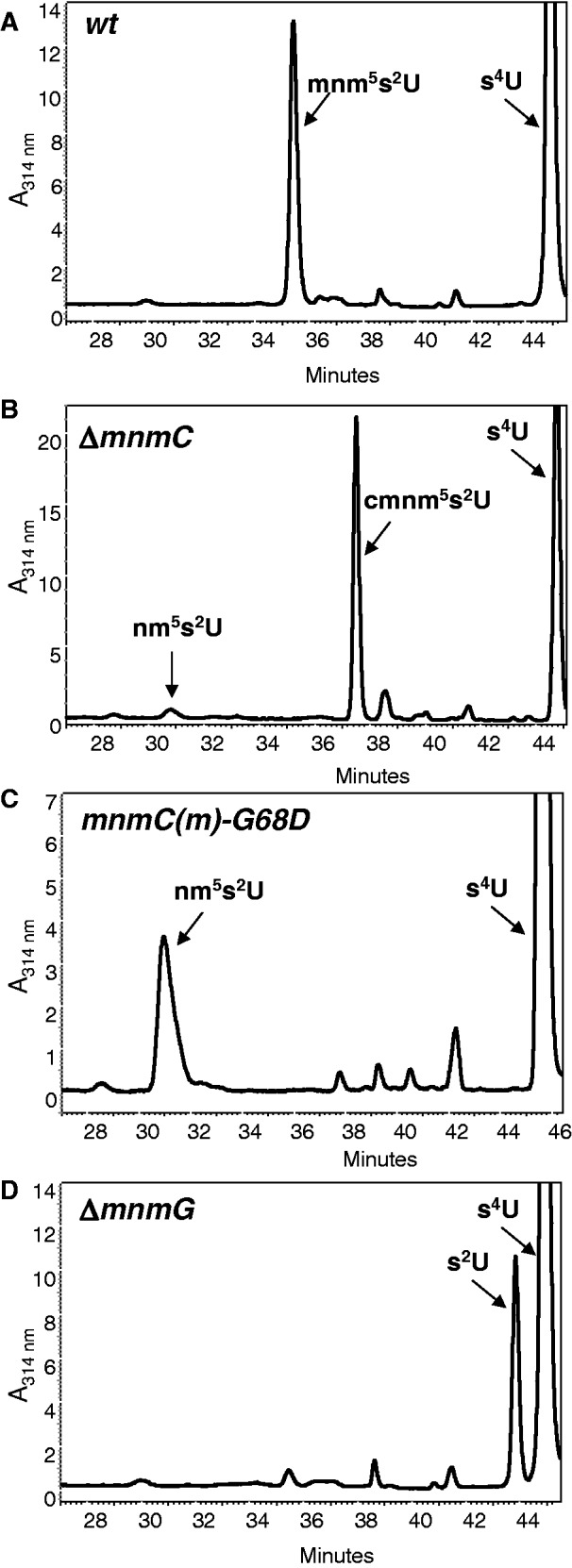
HPLC analysis of native 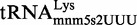 purified from different strains at exponential phase. Native 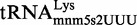 was purified from the wild-type (**A**), *ΔmnmC* (**B**), *mnmC-*G68D (**C**) and *ΔmnmG* (**D**) strains and subjected to HPLC analysis, which was monitored at 314 nm. The relevant nucleosides at position 34 (mnm^5^s^2^U, s^2^U, cmnm^5^s^2^U and nm^5^s^2^U) and position 8 (s^4^U) of 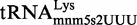 are indicated.

Therefore, in order to gain information on the efficiency of the mnm^5^s^2^U assembly-lines *in vivo*, we decided to explore the modification status of the native 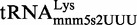 in several strains. The presence of modification intermediates in the tRNA purified from a wild-type strain would be indicative of the MnmEG and MnmC activities not being kinetically tuned. HPLC analysis of native 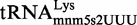 purified during the exponential phase revealed no accumulation of the cmnm^5^s^2^U intermediate in the wild-type strain (Figure 4A), whereas this nucleoside was predominant in the tRNA extracted from the Δ*mnmC* strain (Figure 4B). Moreover, we observed no nm^5^s^2^U in the native 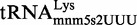 purified from the wild-type strain (Figure 4A), despite this intermediate accumulated in 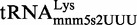 of the *mnmC(m)*-G68D mutant (Figure 4C). These results suggest that 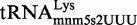 containing cmnm^5^s^2^U or nm^5^s^2^U at the wobble position is stable, and consequently that the disappearance of these intermediates in the wild-type strain is probably due to their conversion into mnm^5^s^2^U by MnmC activities. Thus, it appears that MnmEG and MnmC are kinetically tuned to produce only the final mnm^5^ group in 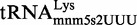.

Notably, the intermediate s^2^U, resulting from the activity of MnmA (Figure 1), was only observed in a *ΔmnmG* strain, which is defective in the MnmEG activity (Figure 4D). Moreover, we did not detect the non-thiolated intermediates cmnm^5^U and nm^5^U when 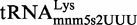 hydrolysates were monitored at 254 nm (data not shown). These data suggest that the coordination of the MnmA- and MnmEGC-dependent pathways is tightly coupled to synthesize mnm^5^s^2^U on native 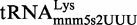 without a build-up of intermediates.

### tRNA substrate specificity of MnmC(o) and MnmC(m) *in vivo*

*Escherichia coli* 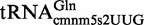 and 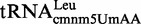 contain cmnm^5^ at the wobble uridine instead of the mnm^5^ found in the remaining MnmEG substrates, (http://modomics.genesilico.pl/sequences/list/tRNA). These data suggest that 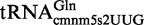 and 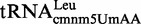 are not substrates for MnmC(o). Moreover, they raise the question of what happens with the ammonium pathway of the MnmEG complex, which theoretically produces nm^5^U in all its tRNA substrates (Figure 1). Interestingly, the analysis of gln1 tRNA from *Salmonella enterica* serovar Typhimurium indicated that 80% of the molecules contained cmnm^5^s^2^U, whereas the remaining 20% contained mnm^5^s^2^U (44). In this case, we speculate that mnm^5^s^2^U might be synthesized by MnmC [using the MnmC(m) activity] from the nm^5^s^2^U generated via the ammonium-dependent MnmEG pathway. Therefore, we decided to investigate whether a fraction of 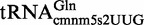 and 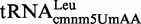 in *E. coli* contains mnm at position 5 in the wobble uridine, and whether both tRNAs function as substrates for MnmC(m) but not MnmC(o) (Figure 1).

To address this question, we constructed a series of tRNA expression plasmids by inserting the coding sequences of 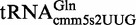, 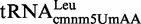 and 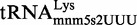 into EcoRI/PstI-digested pBSKrna (37); thus, we eliminated the tRNA scaffold-encoding region (i.e. the DNA region encoding the human cytoplasmic 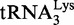) present in the vector (Supplementary Figure S2). Selected strains were transformed with the resulting plasmids. We then examined the HPLC profile of the overexpressed, ‘native’ tRNAs purified from cultures grown overnight to the stationary phase. It should be noted that the expression of the tRNA genes in these constructs was under the control of the strong constitutive *lpp* promoter (37). As mutations leading to reduced expression might be selected over time because overexpression ultimately slows growth, the fresh transformation of cells with the pBSKrna derivatives every time and overnight incubation of the transformed cells prior to the purification of the cloned target (in this case, tRNAs) are highly recommended (37).

**Figure 5.**
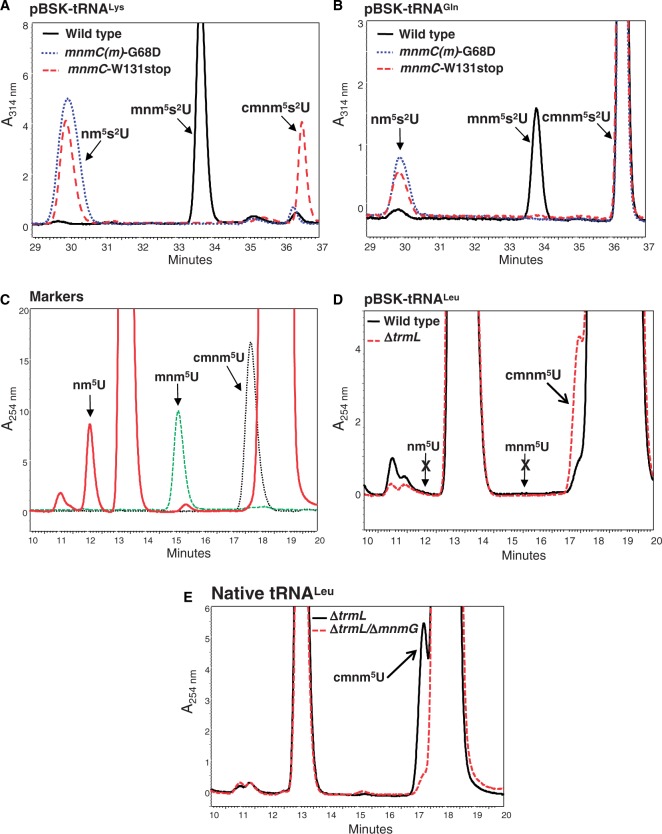
*In vivo* specificity of MnmC(o) and MnmC(m) for substrate tRNAs. Representative HPLC profiles of 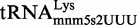 (**A**), 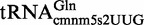 (**B**) and 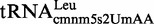 (**D**) expressed from pIC1664, pIC1714 and pIC1665, respectively. The strains used in panels A and B were IC6017 (WT), IC6018 [*mnmC(m)*-G68D] and IC6019 (*mnmC*-W131stop). The strains used in panel D were IC5136 and IC5854. HPLC profiles of commercial markers are shown in panel C. Representative HPLC profiles of native 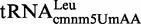 purified from strains *ΔtrmL* (IC6374) and *ΔtrmL/ΔmnmG* (IC6411) are shown (E).

The HPLC analysis of 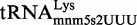 (Figure 5A), used herein as a control, indicated that: (i) mnm^5^s^2^U was predominant in a wild-type strain; (ii) both nm^5^s^2^U and cmnm^5^s^2^U accumulated in the *mnmC* null strain and (iii) nm^5^s^2^U prevailed in the *mnmC(m)*-G68D strain. Taken together, these results indicate that both MnmEG-MnmC(o)-MnmC(m) and MnmEG-MnmC(m) pathways operate on 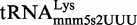 and that they converge to produce the final modification mnm^5^s^2^U in the wild-type strain.

An analysis of overexpressed 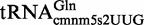 (Figure 5B) indicated that cmnm^5^s^2^U and nm^5^s^2^U accumulated at a ratio of ∼90/10 when tRNA was purified from the *mnmC*-W131stop mutant. Despite the lower proportion of nm^5^s^2^U, the presence of this nucleotide indicates that the MnmEG complex was indeed able to modify 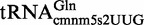 through the ammonium pathway. Nucleosides cmnm^5^s^2^U and mnm^5^s^2^U were found at a 90/10 ratio when 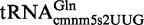 was purified from the wild-type strain. Detection of mnm^5^s^2^U in 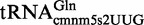 clearly indicates that this tRNA is a substrate for MnmC(m).

It should be noted that both cmnm^5^s^2^U and nm^5^s^2^U accumulated in 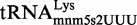 purified from the *mnmC*-W131stop mutant, whereas cmnm^5^s^2^U practically disappeared when 
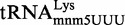
 was purified from the *mnmC(m)*-G68D strain (Figure 5A). This result was expected, considering that cmnm^5^s^2^U was transformed into nm^5^s^2^U by the MnmC(o) activity present in the *mnmC(m)*-G68D strain. Interestingly, the HPLC profiles of 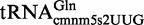 purified from the *mnmC(m)*-G68D and *mnmC*-W131stop strains were similar (Figure 5B). In both cases, cmnm^5^s^2^U was much more abundant than nm^5^s^2^U, suggesting that the MnmC(o) activity present in the *mnmC(m)*-G68D strain did not function in 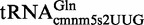.

Overexpression of tRNAs often causes hypomodification. In fact, the hypomodified nucleoside s^2^U, resulting from the action of MnmA, was relatively abundant in the overexpressed 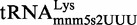 and 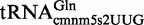 (data not shown). Nevertheless, s^2^U accumulated at a similar proportion in all tested strains so that the ratios of the nucleosides of interest (mnm^5^s^2^U, cmnm^5^s^2^U and nm^5^s^2^U) were not greatly affected. When the 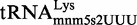 and 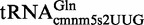 hydrolysates were monitored at 254 nm, the non-thiolated nucleosides nm^5^U, mnm^5^U, cmnm^5^U were hardly detected (data not shown). Therefore, we think that the different HPLC pattern of 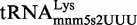 and 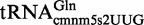 in the *mnmC(m)*-G68D strain cannot be attributed to the presence of hypomodified nucleosides. Rather, it reveals that 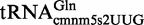 is not a substrate for MnmC(o).

We also analyzed the HPLC profile of 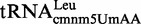 purified from a *trmL* strain in which the methylation of the ribose in the wobble uridine is impaired due to the Δ*trmL* mutation (21). We adopted this approach to examine the modification of 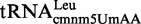 because we were unable to distinguish nucleosides cmnm^5^U_m_, nm^5^U_m_ and mnm^5^U_m_ in our HPLC chromatograms. However, we were able to identify the peaks corresponding to cmnm^5^U, nm^5^U and mnm^5^U using proper markers (Figure 5C). As shown in Figure 5D, overexpressed 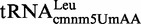 purified from a Δ*trmL* strain contained cmnm^5^U. However, no traces of mnm^5^U or nm^5^U were detected in this tRNA. Similar results were obtained when analyzing native, non-overexpressed 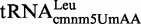 purified in the exponential phase (Figure 5E). Altogether, these data suggest that 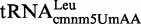 is not modified by the ammonium-dependent MnmEG pathway and that it is not a substrate for MnmC(o).

### tRNA substrate specificity of MnmC(o) and MnmC(m) *in vitro*

**Figure 6.**
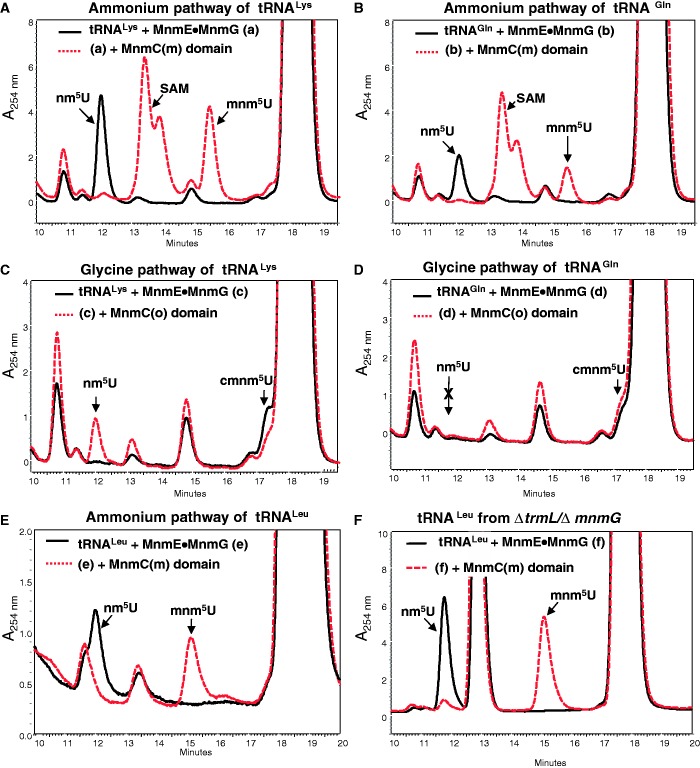
*In vitro* specificity of MnmC(o) and MnmC(m) for substrate tRNAs. (**A–D**) The modification reactions were performed using *in vitro* synthesized tRNAs in two steps. First, the substrate tRNA was modified by the MnmEG complex via the ammonium (A) and (**B**) or the glycine pathway (**C**) and (**D**) and the resulting tRNA (solid black line) carrying nm^5^U or cmnm^5^U was used as a substrate to examine the activity of the MnmC(m) or MnmC(o) domain (dashed red line), respectively. (**E**) HPLC analysis of *in vitro* synthesized 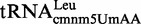 after *in vitro* modification by MnmEG (solid black line) and MnmC(m) (dashed red line). (**F**) Overexpressed 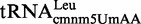 purified from the strain IC6411 (*ΔtrmL/ΔmnmG*) served as the substrate in the ammonium-dependent reaction catalyzed by MnmEG. The resulting tRNA (black line) was used as a substrate for the MnmC(m)-mediated reaction (red line).

To further explore the specificity of MnmC(o) and MnmC(m) for 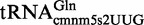, 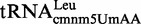 and 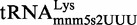, we performed *in vitro* modification reactions using *in vitro* synthesized tRNAs as substrates. These modification reactions included two steps. First, substrate tRNA was modified by the MnmEG complex through the ammonium (Figure 6A, B, E and F) or the glycine (Figure 6C and D) pathways. The resulting tRNA carrying nm^5^U or cmnm^5^U (solid black lines) was subsequently used as a substrate to characterize the activity of the MnmC(m) or the MnmC(o) domains, respectively.

The data in Figure 6 indicate that MnmC(m) catalyzed the conversion of nm^5^U into mnm^5^U in both 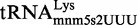 (panel A) and 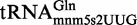 (panel B), whereas MnmC(o) catalyzed the conversion of cmnm^5^ into nm^5^ in 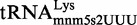 (panel C), but not in 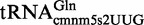 (panel D). These results support the idea that 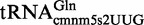 does not function as a substrate for MnmC(o).

Surprisingly, we observed that MnmEG catalyzed the formation of nm^5^U from *in vitro* synthesized 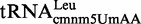 and, in turn, nm^5^U was converted into mnm^5^U through MnmC(m) (Figure 6E). This result contradicts the data provided in Figure 5D and E, where no traces of nm^5^U or mnm^5^U were observed in the HPLC analysis of the 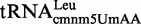 purified from a *trmL* strain. Considering that the experiments shown in Figure 6E were performed using an *in vitro* synthesized 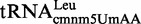 (therefore lacking modifications), we thought that the presence of some modified nucleoside(s) in the *in vivo* synthesized 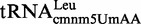 (Figure 5D and E) could hinder the recognition of this substrate by MnmEG. However, when we used overexpressed 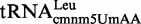 purified from a double *ΔtrmL/ΔmnmG* mutant as a substrate in the *in vitro* modification assay, the MnmEG-mediated synthesis of nm^5^s^2^U was once again observed (Figure 6F). Moreover, the MnmEG-modified 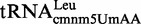 was a good substrate for the *in vitro* synthesis of mnm^5^ through MnmC(m) (Figure 6F). Altogether, these results suggest that the ammonium pathway of MnmEG is ineffective on 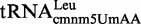 *in vivo*, even though it efficiently functions on this tRNA in the *in vitro* assay.

We also assessed the activity of MnmC(o) on the 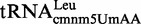 purified from a *trmL* strain and on the *in vitro* transcribed 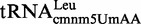 previously modified by MnmEG *in vitro* via the glycine pathway (i.e. on tRNA molecules carrying cmnm^5^U). The synthesis of nm^5^ from cmnm^5^ was not observed under any condition (data not shown). Therefore, we concluded that 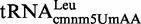 is a substrate *in vitro* for MnmC(m) (Figure 6E and F) but not MnmC(o).

Notably, MnmEG also catalyzed the ammonium-dependent synthesis of nm^5^U in 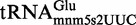, 
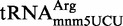
 and 
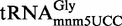
 obtained by *in vitro* transcription (Supplementary Figure S4). We suspect that modification of these tRNAs follows a pattern similar to that observed in 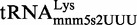, because all these tRNA species appear to contain mainly the mnm^5^ group of U34. However, this hypothesis should be examined in future studies.

### Synthesis of nm^5^U and cmnm^5^U is modulated by growth conditions and the tRNA species

The overexpressed 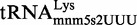 and 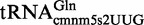 purified from the *mnmC*-W131stop mutant during the stationary phase exhibited different cmnm^5^s^2^U/nm^5^s^2^U ratios (∼35/65 and ∼90/10, respectively; Figure 5A and B). Moreover, the ratio in the overexpressed 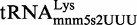 (∼35/65) differed from that found in the total tRNA obtained from the *mnmC*-W131stop strain during exponential growth (∼69/31; Table 2). These results suggest that the glycine pathway (which produces cmnm^5^s^2^U) could be less effective on 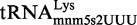 in the stationary phase. These observations prompted us to carefully investigate the efficiency of the glycine and ammonium pathways in total tRNA and specific native tRNAs during the growth curve.

**Figure 7.**
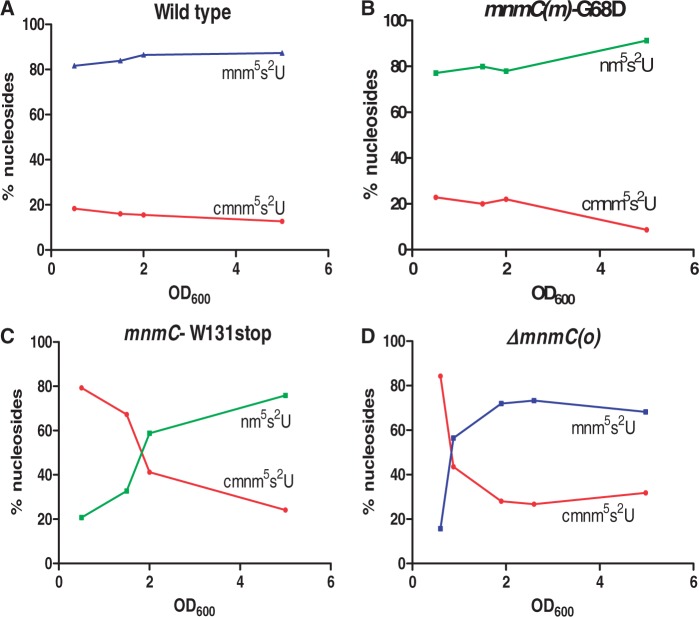
Synthesis of nm^5^U_34_ and cmnm^5^U_34_ is depending on the growth phase. HPLC analysis of total tRNA purified from TH48 (**A**), TH49 (**B**), TH69 (**C**) and IC6629 (**D**) during the growth cycle. The strains were grown in LBT. The percentage of nucleosides represents the distribution of the peak area of each nucleoside compared to the sum of the peak areas of the two nucleosides considered. Each time point represents the average from two independent experiments. The standard deviations were within ±20%.

First, we determined the modification status of total tRNA in the wild-type strain and *mnmC* mutants. In the wild-type or *mnmC(m)*-G68D strain (Figure 7, panels A and B), we could not identify which pathway contributes to the synthesis of the final modification (mnm^5^s^2^U or nm^5^s^2^U, respectively) because the activity of MnmC(o) converges both pathways by transforming cmnm^5^s^2^U into nm^5^s^2^U (Figure 1). However, the strain carrying an *mnmC*-W131stop or *mnmC(o)* null mutation (Figure 7, panels C and D) revealed that the level of cmnm^5^s^2^U and nm^5^s^2^U or mnm^5^s^2^U in bulk tRNA depends on the growth phase; nm^5^s^2^U (or mnm^5^s^2^U) accumulates as optical density increases, whereas cmnm^5^s^2^U exhibits the opposite trend and becomes the minor component at an OD_600_ of ∼2.0. As the data in Figure 7 represent the relative distribution of each nucleoside with respect to the sum of the peak areas of the two nucleosides, it is important to point out that the net decrease in the cmnm^5^s^2^U peak area along the growth curve was concomitant with the net increase in the nm^5^s^2^U or mnm^5^s^2^U area.

Interestingly, when the *ΔmnmC(o)* strain was grown in minimal medium instead of LBT, cmnm^5^s^2^U was consistently observed as the major intermediate, irrespective of the growth phase (Table 4). Therefore, the growth conditions (growth medium and growth phase) affect the pathway responsible for modification at position 5 of U34.

**Table 4.** Reprogramming of the U34 modification depends on the growth conditions

Nucleoside distribution^a^	OD_600 nm_ (LBT)					OD_600 nm_ (MM)		
	0.6	0.9	2	2.6	5 (O/N)	0.6	1.7	3 (O/N)
cmnm^5^s^2^U	86 ± 3	41 ± 4	23 ± 7	22 ± 7	32 ± 1	89 ± 1	96 ± 1	100
mnm^5^s^2^U	14 ± 3	59 ± 4	77 ± 7	78 ± 7	68 ± 1	11 ± 1	4 ± 1	0

^a^Relative distribution (%) of nucleosides. Total tRNAs were purified throughout the growth cycle from strain *ΔmnmC*(*o*) (IC6629) growing in LBT or minimal medium (MM; YM9 supplemented with 0.4% glucose) and analyzed by HPLC. The OD_600_ of the overnight (O/N) cultures was different according to the growth medium.

We subsequently analyzed the behavior of native tRNAs in both the exponential and stationary phases (Table 5 and Supplementary Figure S5). During the exponential growth of the *ΔmnmC* strain, cmnm^5^s^2^U was the major intermediate accumulated in native 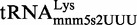 (94%), but this nucleoside became the minor intermediate (4%) in the stationary phase. In contrast, HPLC analysis revealed that cmnm^5^s^2^U was consistently the major intermediate present in native 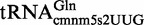 (>90%), irrespective of the growth phase. Altogether, these results indicate that 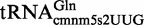 functions different from 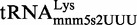 and total tRNA when the *ΔmnmC* strain is grown to stationary phase in LBT (i.e. in growth conditions that favor the ammonium pathway). Therefore, the accumulation of cmnm^5^ or nm^5^ depends on the specific characteristics of the tRNA.

**Table 5.** Reprogramming of U34 modification also depends on the tRNA species

Specific tRNAs	Growth Phase	LBT^a^	
		cmnm^5^s^2^U	nm^5^s^2^U
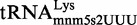	Exponential	94 ± 1	6 ± 1
	Stationary	4 ± 2	96 ± 2
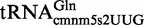	Exponential	99 ± 1	1 ± 1
	Stationary	93 ± 2	7 ± 2

^a^Relative (%) distribution of the nucleosides in accordance with the growth phase and the tRNA species. Native tRNAs were purified from strain *ΔmnmC* (IC6010) during the exponential (OD_600_ ∼0.4) or stationary phase (OD_600_ ∼3).

### Importance of MnmC-mediated modifications for cell growth

The limited data available regarding the effect of *mnmC* mutations on growth rate are contradictory. No differences between *mnmC* and wild-type strains were initially reported (22,45). However, more recently, Pearson and Carell (26) measured the growth rate of an *mnmC*-knockout strain from the Keio collection and observed a larger growth constant for the wild-type strain, in agreement with the hypothesis that accumulation of intermediates in the synthesis of mnm^5^(s^2^)U affects the translation process negatively. We investigated the effect of *mnmC* mutations on growth rate in different genetic backgrounds and did not observe significant differences between the wild-type strain and any derivative carrying an *mnmC* allele [*ΔmnmC*, *mnmC*-W131stop, *ΔmnmC(o)* and *mnmC(m)*-G68D; Table 6]. We cannot explain why our results differ from those reported by Pearson and Carell (26), even when the same background (BW25113) was used. Perhaps, the dilution method used to maintain the pre-cultures in exponential growth could explain the different results. We took great care to not dilute the pre-cultures below an OD_600_ of 0.25 and to not exceed an OD_600_ of 0.5 in order to avoid major changes under the exponential growth conditions.

**Table 6.** Growth rates of *mnmC* mutants in different genetic backgrounds

Background	Doubling time (min)**^a^**							
	wt	*mnmC*^b^	*mnmC(m)*^c^	*ΔmnmC(o)*	*ΔmnmA*	*mnmA*	*ΔmnmA*	*ΔmnmA*
						*ΔmnmC*^d^	*mnmC(m)*^c^	*ΔmnmC(o)*
TH48	34 ± 1	33 ± 1	32 ± 1	nd	nd	nd	nd	nd
BW25113	28 ± 1	30 ± 1	nd	28 ± 1	40 ± 1	nd	nd	58 ± 3
IC4639	28 ± 1	29 ± 1	30 ± 1	nd	56 ± 1	73 ± 2	78 ± 2	nd

^a^Doubling-time values of strains (wild-type or carrying the indicated mutations) are the mean ± SD of at least two independent experiments.

^b^The null *mnmC* alleles were *mnmC*-W131stop (TH48 background) and *ΔmnmC* (IC4639 and BW25113 backgrounds).

^c^The *mnmC(m)* point mutation was *mnmC(m)*-G68D. nd, not done.

^d^The mnmA point mutation was *mnmA*-Q233stop.

**Figure 8.**
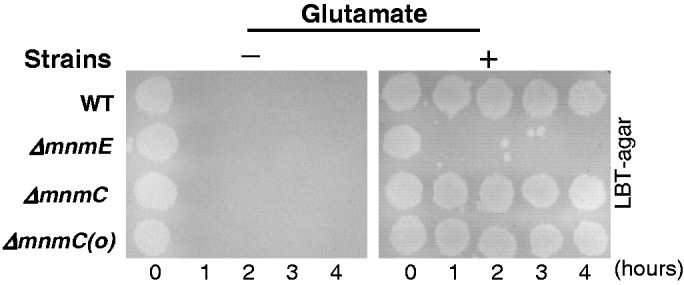
The effect of *mnmE* and *mnmC* mutations on acid resistance. Strains IC5136 (wild-type), IC5827 (*ΔmnmE*), IC6010 (*ΔmnmC*) and IC6629 [*ΔmnmC*(*o*)] were grown in LBT containing 0.4% glucose to stationary phase and then diluted 1:1000 into EG pH 2.0 medium supplemented or not with glutamate. The samples were spotted after 0, 1, 2, 3 and 4 h post acid challenge on LAT plates.

The lack of effect in our hands of *mnmC* mutations during the exponential growth of cells contrasts with the behavior of *mnmE*- and *mnmG*-knockout strains, which, as we have previously demonstrated, grow slower than the wild-type strain (12,32). These data suggest that the lack of any modification at the wobble uridine is more deleterious to the cell than the presence of any intermediate of mnm^5^(s^2^) synthesis. Supporting this conclusion, we also observed that the *mnmC* mutations, unlike the *mnmE* mutations, did not affect resistance to acid stress of *E. coli* (Figure 8). This resistance depends on several systems, one of which involves proteins whose translation likely depends on tRNAs modified by MnmEG (39,46).

The MnmA protein catalyzes the last step of thiolation at position 2 of U34 in 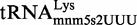, 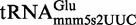, and 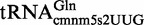 (Figure 1). Inactivation of the *mnmA* gene has been reported to increase the doubling time (31,47). Interestingly, combination of the *mnmA* and *mnmC* mutations had a synergic effect (Table 6), revealing that loss of any MnmC activity is detrimental under specific conditions. Mutation *mnmC(m)*-G68D appeared to slow down the growth rate of the *mnmA*-knockout strain slightly more than the *ΔmnmC* mutation.

We also performed competition experiments between BW25113 (which is a wild-type, tetracycline-sensitive strain) and TH48 (wild-type), TH49 (*mnmC(m)*-G68D) and TH69 (*mnmC*-W131stop), which are tetracycline-resistant strains (Table 7). This type of experiment compares not only the growth in exponential phase, but also the ability to sustain and recover from stationary phase. Although TH48 and its derivatives were out-competed by BW25113, it is clear that the impairment of the MnmC functions reduces the ability of the strain to compete. Curiously, in these experiments, mutant *mnmC*-W131stop was less competitive than *mnmC(m)*-G68D.

**Table 7.** Growth competition assay

Strain^a^	Ratio^b^ at mix time	Ratio^b^ after six 24-h cycles
Wild-type (TH48)	0.56 ± 0.007	0.021 ± 0.003
*mnmC(m)*-G68D (TH49)	0.53 ± 0.09	0.0020 ± 0.0006
*mnmC-*W131stop (TH69)	0.56 ± 0.04	0.0002 ± 0.0001

^a^BW25113 (tetracycline-sensitive strain) and the indicated TH strain (tetracycline-resistant strain) were mixed 1:1 at the start of the experiment.

^b^The ratio was calculated as CFU/ml recovered on LAT supplemented with tetracycline versus CFU/mL recovered on LAT.

## DISCUSSION

This study aimed to further explore the activities and specificities of the MnmEG complex and the MnmC domains, the ability of specific tRNAs to follow the ammonium- or glycine-MnmEG pathway under different growth conditions and the effect of *mnmC* mutations on cell growth.

The fulfillment of the two first aims was facilitated by the cloning and separate expression of the MnmC domains. We demonstrate for the first time that the MnmC(o) domain can fold independently of MnmC(m) and that both domains are catalytically active when expressed separately. Importantly, the domains exhibit similar kinetic properties to those observed in the entire MnmC protein, which suggests that the two domains operate independently of each other. Moreover, MnmC(o) and MnmC(m) differ in terms of their specificity for tRNA substrates. Thus, MnmC(o) modifies 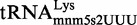, but not 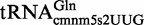 and 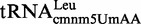. In contrast, all three tRNAs (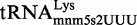, 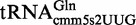 and 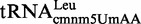) serve as substrates for MnmC(m) not only *in vitro*, but also *in vivo* at least in the cases of 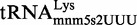 and 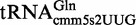. These data strongly support the notion that the MnmC(o) and MnmC(m) domains function independently. The presence of mnm^5^s^2^U in *in vivo* synthesized 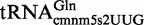 suggests that the nomenclature of this tRNA should be changed to 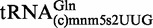.

Sequence searches of complete genomes have revealed that the distribution of the putative orthologs of the *E. coli* bi-functional MnmC protein are conserved only in γ-proteobacteria, with some additional members in other bacterial classes (24). Interestingly, in several genomes, potential orthologs of a single domain have been identified (24), but the biological function has been experimentally demonstrated only in the case of the *Aquifex aeolicus* DUF752 protein, an MnmC(m) homolog (41). It is unclear from the phylogenetic analysis whether the independent putative orthologs represent the ancestral or derived versions of the bi-functional MnmC enzyme (24). The ability of the *E. coli* MnmC(o) and MnmC(m) domains to function independent of each other, as shown in this work, suggests that the origin of the full MnmC protein present in γ-Proteobacteria (24) likely occurred by domain fusion. The MnmC(o)–MnmC(m) fusion in *E. coli* suggests a functional advantage of the spatial proximity of both domains. The fusion could increase the stability of the resulting protein. In fact, our results indicate that the entire MnmC protein is substantially more stable than the separate domains (Figure 2C) or other RNA modification proteins, such as RsmG or MnmG, which have half-lives of ∼17 and 31 min, respectively (48). The possibility that the fusion also facilitates the functional cooperation between the domains during modification of tRNAs following the ‘canonical’ MnmEG-MnmC(o)–MnmC(m) pathway (cmnm^5^U→nm^5^U→mnm^5^U) cannot be ruled out. A better understanding of how the MnmC protein functions will entail determining whether the binding of a tRNA molecule to one domain exerts a negative allosteric effect on the other domain. In this respect, the structural characterization of MnmC–tRNA complexes could prove helpful.

Biosynthesis of complex modifications like mnm^5^s^2^U, cmnm^5^s^2^U, cmnm^5^Um and mnm^5^U requires the participation of at least two specific enzymes (Figure 1). Our *in vivo* data suggest that MnmEG and MnmC activities are kinetically tuned to produce the final modification mnm^5^s^2^U on native 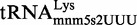 because no intermediates (cmnm^5^s^2^U and nm^5^s^2^U) were observed in a wild-type strain (Figure 4). Apparently, the intermediates are not subject to active degradation as they were shown to be stable in strains *ΔmnmC* and *mnmC(m)*-G68D. Notably, neither s^2^U nor the non-thiolated nucleosides cmnm^5^U and nm^5^U were found in native 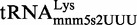 purified from the wild-type strain (Figure 4; data not shown). Therefore, we believe that the MnmEG–MnmC and MnmA pathways are also coordinated by tuning the activities and relative abundances of the tRNA-modifying enzymes. Modifications of U34 by MnmEG and MnmA occur independently of each other [(30,49); Figure 4D], but whether the presence of the s^2^ group facilitates the modification of position 5 in 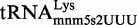, 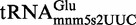 and 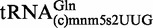 by modulating the electron density distribution in the uridine ring remains unclear.

There are very few data available on the identity determinants required by TrmL. Methylation of the ribose 2′-OH group by this enzyme occurs as a late step in 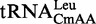 maturation given that the enzyme fails to methylate 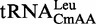 without the prior addition of *N*^6^-(isopentenyl)-2-methyladenosine (ms^2^i^6^A) at position 37 (21). Nucleoside ms^2^i^6^A is also present in 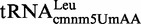. Hence, it is feasible that TrmL also requires ms^2^i^6^A as a positive identity determinant to modify 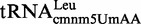. Here our data indicate that MnmEG modifies 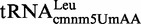 in the absence of 2′-*O*-methylation (Figures 5D and E; 6E and F), but we do not know whether TrmL requires the presence of the cmnm^5^ group to act on 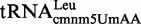. More experiments are needed to unravel the order of action of the modifying enzymes working on U34 and the identity elements required by each enzyme.

Another interesting question concerning tRNA maturation is whether physical interactions among tRNA modification enzymes contribute to the efficiency of complex pathways. In *Aquifex aeolicus*, an interaction between the *E. coli* MnmC(m) homolog, protein DUF752 and the *A. aeolicus* MnmE and MnmG proteins has been observed (41). In *E. coli*, we did not detect interaction of MnmC with MnmE or MnmG by either fast-performance liquid chromatography (FPLC) analysis (Figure 2F) or SPR (data not shown). However, we cannot rule out the possibility of the MnmEG complex being able to interact with MnmC *in vivo*. It appears reasonable to hypothesize that the spatial clustering of tRNA-modifying enzymes within the cell can optimize the coordination of their work. Therefore, one challenging aim for future studies is to determine whether tRNA modifying enzymes are spatially organized, as observed for other enzymes involved in RNA biology, such as the components of the RNA degradosome (50).

**Figure 9.**
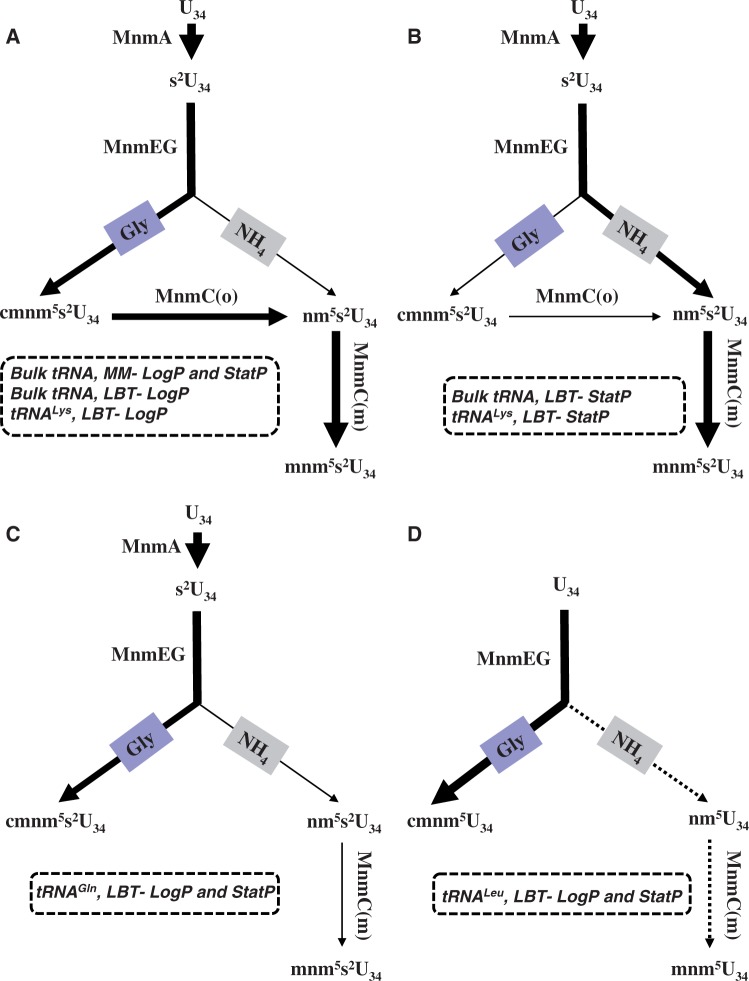
Summary scheme of the reprogramming of U34 base modification in both bulk and specific tRNAs. The main and minor modification pathways are indicated by thick and thin arrows, respectively; pathways whose activity was only observed *in vitro* are indicated by dotted arrows. Activity of TrmL on 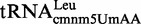 has been omitted in panel D. Labels in each panel indicate the type of tRNA (specific or bulk tRNA) following the pathways, and the growth conditions under which the tRNA was obtained. LogP, logarithmic phase; StatP, stationary phase; MM, minimal medium; LBT, LBT medium.

The use of strains carrying the *ΔmnmC(o)* or *ΔmnmC* mutation has allowed us to study the ability of MnmEG to use the ammonium or the glycine pathway under different growth conditions and thus, to detect the reprogramming of U34 base modification in both bulk and specific tRNAs (Tables 4 and 5; Figure 9). The analysis of bulk tRNA indicates that when *ΔmnmC(o)* or *ΔmnmC* strains are grown in LBT, the glycine pathway prevails over the ammonium pathway in the exponential phase as cmnm^5^s^2^U is the most abundant nucleoside (Figure 9A). However, a gradual change in the output of both pathways is observed along the growth curve so that the nucleosides produced by the ammonium pathway become prevalent during entry into the stationary phase (Figure 9B). In contrast, when cells are grown in minimal medium, the glycine pathway is prevalent in both the exponential and stationary phases (Figure 9A). A preference by MnmEG for the ammonium or the glycine pathway to catalyze tRNA modification may depend on the availability of these substrates, which, in turn depends on the growth medium and cell metabolism.

The nature of tRNA species also appears crucial for the net output of the MnmEG pathways (Table 5 and Figure 9). Thus, whereas 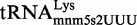 follows the pattern observed in bulk tRNA and is preferably modified via the ammonium pathway during the transition to the stationary phase, 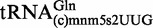 fails to be effectively modified through this pathway, as indicated by the low accumulation of nm^5^s^2^U in the *ΔmnmC* mutant. Moreover, the ammonium pathway proves ineffective in modifying 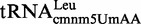 at any phase of growth as no nm^5^U or mnm^5^U is detected in a *trmL* strain (Figure 5D and E). Therefore, neither 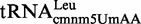 nor 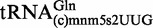 is a good substrate for the ammonium pathway *in vivo*, even though both tRNAs appear rather well modified by this pathway *in vitro* (Figure 6B and E; Figure 9C and D).

Why the behavior of 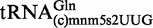 and 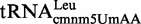 is different from that of 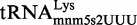 remains unresolved. The use of ammonium or glycine by MnmEG could be regulated by interactions of this complex with its tRNA substrates and unknown factors *in vivo*. 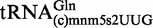 and 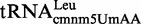 may be poor substrates for the ammonium pathway and thus out-competed by other tRNAs *in vivo*. Alternatively, 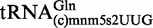 and 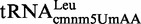 carrying nm^5^ or mnm^5^, but not cmnm^5^ may be unstable and specifically degraded by some nuclease(s) (5,51). Additional experiments are required to test these hypotheses and to elucidate the mechanism underlying the selection by MnmEG of the glycine or the ammonium pathway.

Our results highlight the crucial role of MnmC(o) and Mnm(m) in the biosynthesis of mnm^5^(s^2^)U in accordance with growth conditions (Figure 9). During the exponential phase in LBT and at any phase in minimal medium, the oxidoreductase activity of MnmC(o) is required to drive the intermediate generated by the more active glycine pathway [cmnm^5^(s^2^)U] toward the MnmC(m)-dependent synthesis of the final modification in 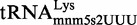 and, presumably, in 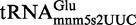, 
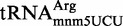
 and 
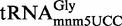
 (Figure 9A). In contrast, MnmC(o) plays a minor role when the ammonium pathway prevails; i.e. during the transition to stationary phase of cultures growing in LBT. In this case, the methylase activity of MnmC(m) functions fairly well alone [i.e. without the participation of MnmC(o)] to drive the production of the final modification mnm^5^(s^2^)U by transforming the intermediate nm^5^(s^2^) generated by the more active ammonium pathway (Table 4 and Figure 9B). Thus, the incorporation of MnmC(o) and MnmC(m) into the enzyme content of *E. coli* extends the adaptability of this bacterium to changing growth conditions. How our findings in *E. coli* apply to other bacteria, especially those lacking certain MnmC activity, is an issue that merits future research.

The lack of modifications at the wobble uridine due to the presence of *mnmE* or *mnmG* mutations leads to impaired growth, high sensitivity to acidic pH and defects in translational fidelity(12,35,46,48,52–55). Here our functional studies suggest that MnmC impairment is less detrimental than the inactivation of the MnmEG complex given that *mnmC* mutations neither reduce the growth rate in rich medium (Table 6) nor affect resistance to acidic pH (Figure 8). Nevertheless, impairment of any MnmC activity has a biological cost. Thus, *mnmC* mutations diminish the ability of cells to compete (Table 7), suggesting that fully modified tRNAs and, therefore the activities of MnmC, are required for efficient translation of mRNAs involved in the stationary phase stress response. In addition, our data indicate that *mnmC* mutations slow the growth rate of a *mnmA* strain, revealing a synergistic effect of mutations *mnmC* and *mnmA*. As *mnmC* mutations alone do not affect the growth rate, we hypothesize that the presence of the MnmA-dependent thiolation at position 2 of U34 in a subset of tRNAs can compensate for the lack of MnmC modifications at position 5 during exponential growth.

In an *mnmA* background, accumulation of nm^5^U produced by the *mnmC(m)*-G68D mutation appears slightly more detrimental than accumulation of cmnm^5^ caused by a null *mnmC* mutation (Table 6). In line with this, it has been demonstrated that the reading of an amber codon by an ochre-suppressing derivative of 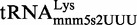 (*supG*) is also more affected by the *mnmC(m)*-G68D mutation than by the null *mnmC*-W131stop mutation, although this effect is dependent on the codon context (56). Therefore, it appears that cmnm^5^(s^2^) confers the cell some advantage over nm^5^(s^2^)U to translate specific mRNAs during exponential growth.

Strikingly, in the competition experiments (Table 7), the *mnmC*-W131stop mutation appears to be more disadvantageous than the *mnmC(m)*-G68D allele, which reveals an opposite trend to that observed in the growth rate determination experiments (Table 6). During the transition to the stationary phase, nm^5^(s^2^)U is more abundant in *mnmC(m)*-G68D than in *mnmC*-W131stop (Figure 7, panels B and C). Therefore, the slightly better ability of the *mnmC(m)*-G68D mutant to compete might indicate that nm^5^(s^2^)U is more effective than cmnm^5^(s^2^)U in translating mRNAs required to sustain stationary phase stress conditions. Thus, it appears that the advantages offered by each intermediate, nm^5^(s^2^)U and cmnm^5^(s^2^)U, depend on the nature of the mRNAs to be translated which, in turn, depend on the growth conditions. Obviously, the synthesis of the final modification mnm^5^s^2^U is more advantageous to the cell as the strains expressing a fully active MnmC protein grow better under stressing conditions (i.e. those related to lack of MnmA and competition with other bacteria) than those strains lacking any MnmC activity. In conclusion, adaptation of MnmEG to use ammonium or glycine, together with the acquisition of MnmC(o) and MnmC(m) activities, provide *E. coli* with significant advantages to synthesize mnm^5^s^2^U and to survive under different conditions.

## SUPPLEMENTARY DATA

Supplementary Data are available at NAR Online.

## FUNDING

The Spanish Ministry of Economy and Competitiveness [BFU2007-66509 and BFU2010-19737]; the Generalitat Valenciana [ACOMP/2012/065; PROMETEO/2012/061 to M.-E.A.]; the Swedish Science Research Council [project BU-2930] and Carl Trygger Foundation (to G.R.B.); a pre-doctoral fellowship from the Spanish Ministry of Economy and Competitiveness (to M.-J.G.); I.M. was supported by short-term fellowships from the Ministry of Science and Innovation and Generalitat Valenciana during his stays at the Umea University. Funding for open access charge: Spanish Ministry of Economy and Competitiveness [BFU2010-19737].

*Conflict of interest statement*. None declared.

## Supplementary Material

Supplementary Data
