# Supplementary material for: The output of the tRNA modification pathways controlled by the Escherichia coli MnmEG and MnmC enzymes depends on the growth conditions and the tRNA species
Source: Nucleic Acids Res. 2013 Nov 26;42(4):2602–23. doi: 10.1093/nar/gkt1228 (PMC3936742; doi:10.1093/nar/gkt1228)
Supplement: Supplementary Data [file supp_42_4_2602__index.html]

The output of the tRNA modification pathways controlled by the Escherichia coli MnmEG and MnmC enzymes depends on the growth conditions and the tRNA species — The output of the tRNA modification pathways controlled by the Escherichia coli MnmEG and MnmC enzymes depends on the growth conditions and the tRNA species — Supplementary Data 

# The output of the tRNA modification pathways controlled by the *Escherichia coli* MnmEG and MnmC enzymes depends on the growth conditions and the tRNA species

## Supplementary Data

files

**Files in this Data Supplement:**

- Supplementary Data - pdf file
