# Supplementary material for: The output of the tRNA modification pathways controlled by the Escherichia coli MnmEG and MnmC enzymes depends on the growth conditions and the tRNA species
Source: Nucleic Acids Res. 2013 Nov 26;42(4):2602–23. doi: 10.1093/nar/gkt1228 (PMC3936742; doi:10.1093/nar/gkt1228)
Supplement: Supplementary Data [file supp_gkt1228_nar-03115-z-2013-File003.pdf]

SUPPLEMENTARY DATA

The output of the tRNA modification pathways controlled by the *Escherichia coli* MnmEG and MnmC enzymes depends on the growth conditions and the tRNA species

Ismaïl Moukadiri[1&]*, M.-José Garzón[1&], Glenn R. Björk[2], M.-Eugenia Armengod[1,3]*

[1]Laboratory of RNA Modification and Mitochondrial Diseases, Príncipe Felipe Research Center, 46012-Valencia, Spain
[2]Department of Molecular Biology, Umeå University, S90187, Sweden
[3]Biomedical Research Networking Centre for Rare Diseases (CIBERER) (node U721), Spain

[&]The authors wish it to be known that, in their opinion, the first two authors should be regarded as joint First Authors

*Corresponding author

Corresponding and submitting author:
M.-Eugenia Armengod
Laboratory of RNA Modification and Mitochondrial Diseases
Príncipe Felipe Research Centre
Eduardo Primo Yúfera St., 3
46012-Valencia, Spain
E-mail: armengod@cipf.es
Phone: 34-963289681 (2006#)
Fax: 34-963289701

Running head: Biochemical and functional study of MnmEG and MnmC

Supplementary Table S1. Oligonucleotides

**To construct the *mnmC(o)* domain deletion in the *E. coli* chromosome**[a]	
**MnmC(o)Δ-F**<br>**MnmC(o)Δ-R**	tgcggggtgatggaacagacattaccgctcccctgctccgcgTAA***TGTAGGCTGGAGCTGCTTCG***<br>TACAATGATGTTGATTCGCCGGATTTTGTGGCGTGAGATAACCG***CATATGAATATCCTCCTTAG***
**To clone the *mnmC* domains**[b]	
**Flag-MnmC(o)F**<br>**Flag-MnmC(o)R**	GACTATAAAGACGACGACGACAAACCCTGCTCCGCGCCGTGGTT<br>*TTA*CCCCGCCTTAACCGCTTTACCCTTCAAC
**Flag-MnmC(m)F**<br>**Flag-MnmC(m)R**	GACTATAAAGACGACGACGACAAAAAACACTACTCCATACAACC<br>*TTA*GAGCGGTAATGTCTGTTCCATCACC
**His-MnmC(m)F**<br>**His-MnmC(m)R**	catcatcatcatcatcacAAACACTACTCCATACAACCTGCC<br>*TTA*GAGCGGTAATGTCTGTTCCATCACC
**To clone specific tRNAs**	
**For insertion into pUC19 (SmaI) or pBAD-TOPO**[c]	
**Leu-F**<br>**Leu-R**	***aagctT***AATACGACTCACTATAGCCCGGATGGTGGAATCGG<br>***aagctT***GGTACCCGGAGCGGGACTTGAACCCGCAC
**Gln-F**<br>**Gln-R**	***aagctT***AATACGACTCACTATAGTGGGGTATCGCCAAGCGG<br>***aagctT***GGCTGGGGTACCTGGATTCGAACCAGGGAATGC
**Glu-F**<br>**Glu-R**	***aagctT***AATACGACTCACTATAGTCCCCTTCGTCTAGAGGCC<br>***aagctT***GGCGTCCCCTAGGGGATTCGAACCCCTG
**Gly-F**<br>**Gly-R**	***AagctT***AATACGACTCACTATAGCGGGCATCGTATAATGGC<br>***AagctT***GGAGCGGGCAGCGGGAATCGAACCCG
**Arg-F**<br>**Arg-R**	gc**ccagg**TAATACGACTCACTATAGCGCCCTTAGCTCAGTTG<br>gc**ccagg**TGGCGCGCCCTGCAGGATTCGAACCTGCGGC
**Cys-F**<br>**Cys-R**	gc**ccagg**TAATACGACTCACTATAGGCGCGTTAACAAAGCGG<br>gc**ccagg**TGGAGGCGCGTTCCGGAGTCGAACCGGAC
**For insertion into pBSKrna(EcoRV)**[d]	
**Lys- EcoRV-F**<br>**Lys- EcoRV-R**	CCG***GATATC***GGGTCGTTAGCTCAGTTGGTAG<br>CCG***GATATC***TGGTGGGTCGTGCAGGATTCG
**For insertion into pBSKrna(EcoR1-Pst1)**[e]	
**Lys-EcoRI**<br>**Lys-PstI**	CCG***GAATTC***GGGTCGTTAGCTCAGTTGGTAGAG<br>TGCA*CTGCAG*TGGTGGGTCGTGCAGGATTCGAAC
**Leu-EcoRI**<br>**Leu-PstI**	CCG***GAATTC***GCCCGGATGGTGGAATCGGTAG<br>TGCA*CTGCAG*TGGTACCCGGAGCGGGACTTGAAC
**Gln-EcoRI**<br>**Gln-PstI**	CCG***GAATTC***TGGGGTATCGCCAAGCGGTAAGG<br>TGCA*CTGCAG*TGGCTGGGGTACCTGGATTCGAACCAGG
**Biotinylated oligos used for purification of specific *E. coli* tRNAs**	
**Lys**	*Biotin*-TGGTGGGTCGTGCAGGATTCGAACCTG
**Leu**	*Biotin*-TGGTACCCGGAGCGGGACTTGAACCC
**Gln**	*Biotin*-GAACCAGGGAATGCCGGTATCAAAAAC
**Biotinylated oligo used for purification of chi-tRNAs**	
**Hum-Cyt-Lys (Scaffold)**	*Biotin*-TGGCGCCCGAACAGGGACTTGAACCC

[a] Sequences in bold correspond to the *cat* cassette. The TAA stop codon is underlined.
[b] *Flag* and *His* tag sequences are underlined. The codon stop TAA (TTA complementary sequence) is in italics.
[c]T7-promoter sequence is underlined, HindIII sites are in italics and in bold, and BstN1 sites are in bold (red)
[d]EcoRV sites are in italics in bold.
[e]EcoRI and PstI sites are in bold and underlined, respectively.

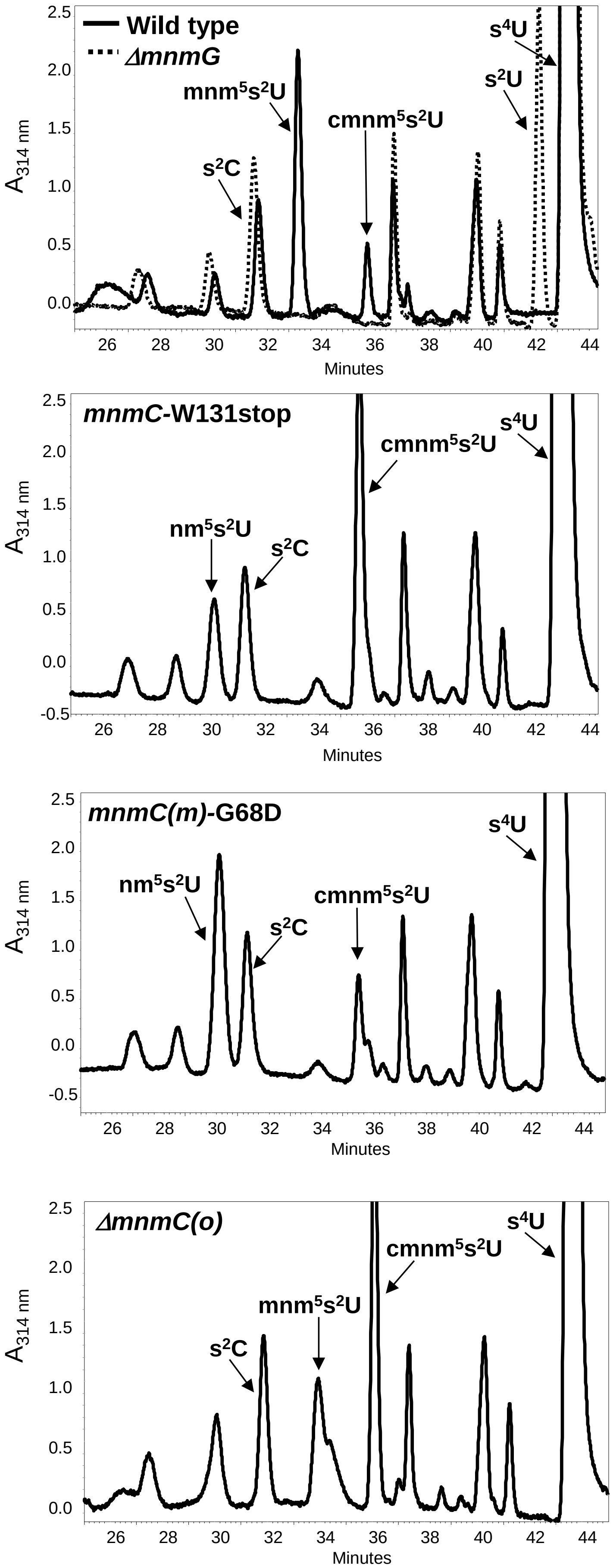

Supplementary Figure S1. Genetic analysis of the functional activities of MnmEG and MnmC. Bulk tRNA was purified from exponentially growing strains with different genotypes (wild-type, *ΔmnmG*, *mnmC*-W131stop, *mnmC(m)*-G68D, and *ΔmnmC(o)*), and subjected to HPLC analysis after treatment with nuclease P1 and alkaline phosphatase. The nucleosides were monitored at 314 nm to maximize the detection of thiolated nucleosides.

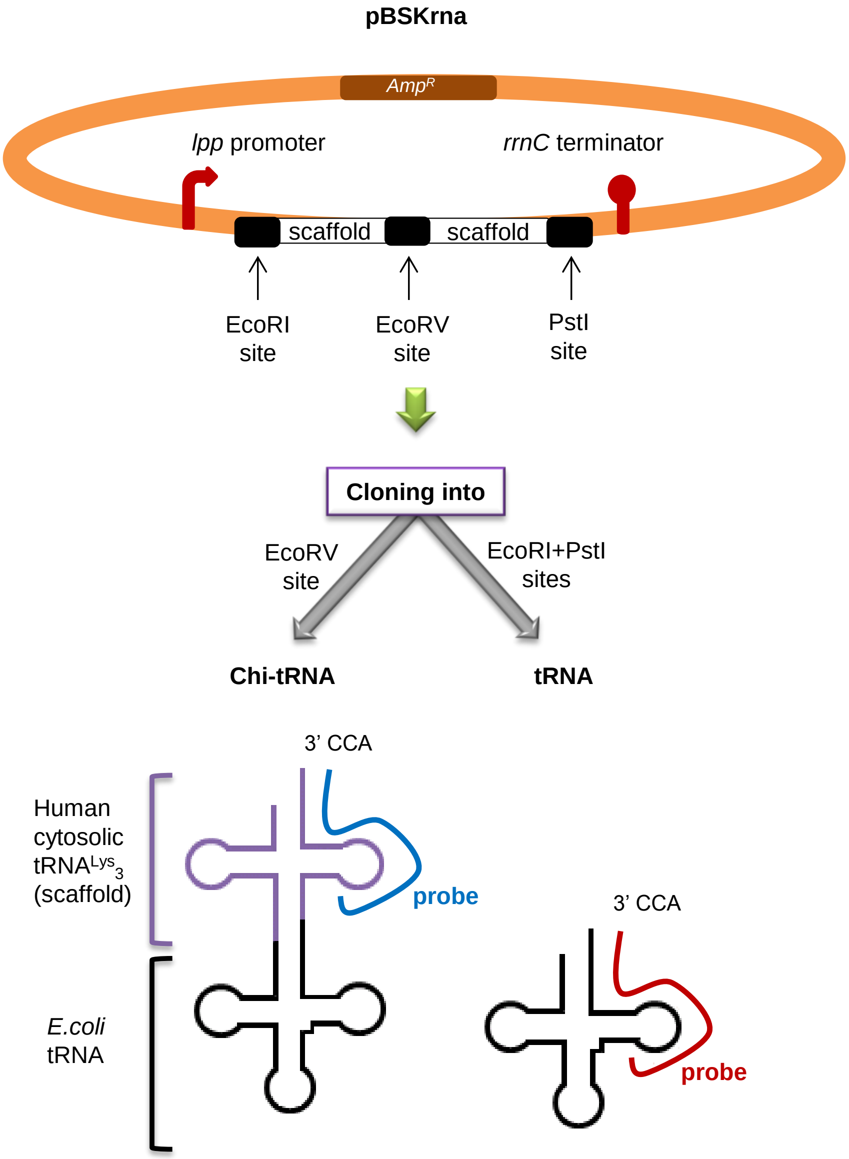

Supplementary Figure S2. General scheme for the construction of tRNA overexpressing plasmids. The expression vector (pBSKrna) contains the strong lipoprotein gene promoter (*lpp* promoter), the ribosomal RNA operon transcription terminator (*rrnC* terminator), and the tRNA scaffold coding gene. The latter is the human cytosolic $tRNA^{Lys}_3$ gene lacking the anticodon region, which has been substituted by a sequence containing the EcoRV cloning site. Digestion of the vector with EcoRI and PstI deletes the tRNA scaffold coding gene, allowing expression of the desired gene under control of the *lpp* promoter and the *rrnC* terminator. Cloning into the scaffold region facilitates the isolation of all chimeric tRNAs with the same probe, whereas cloning after deletion of the scaffold coding gene involves the use of probes specific for each gene.

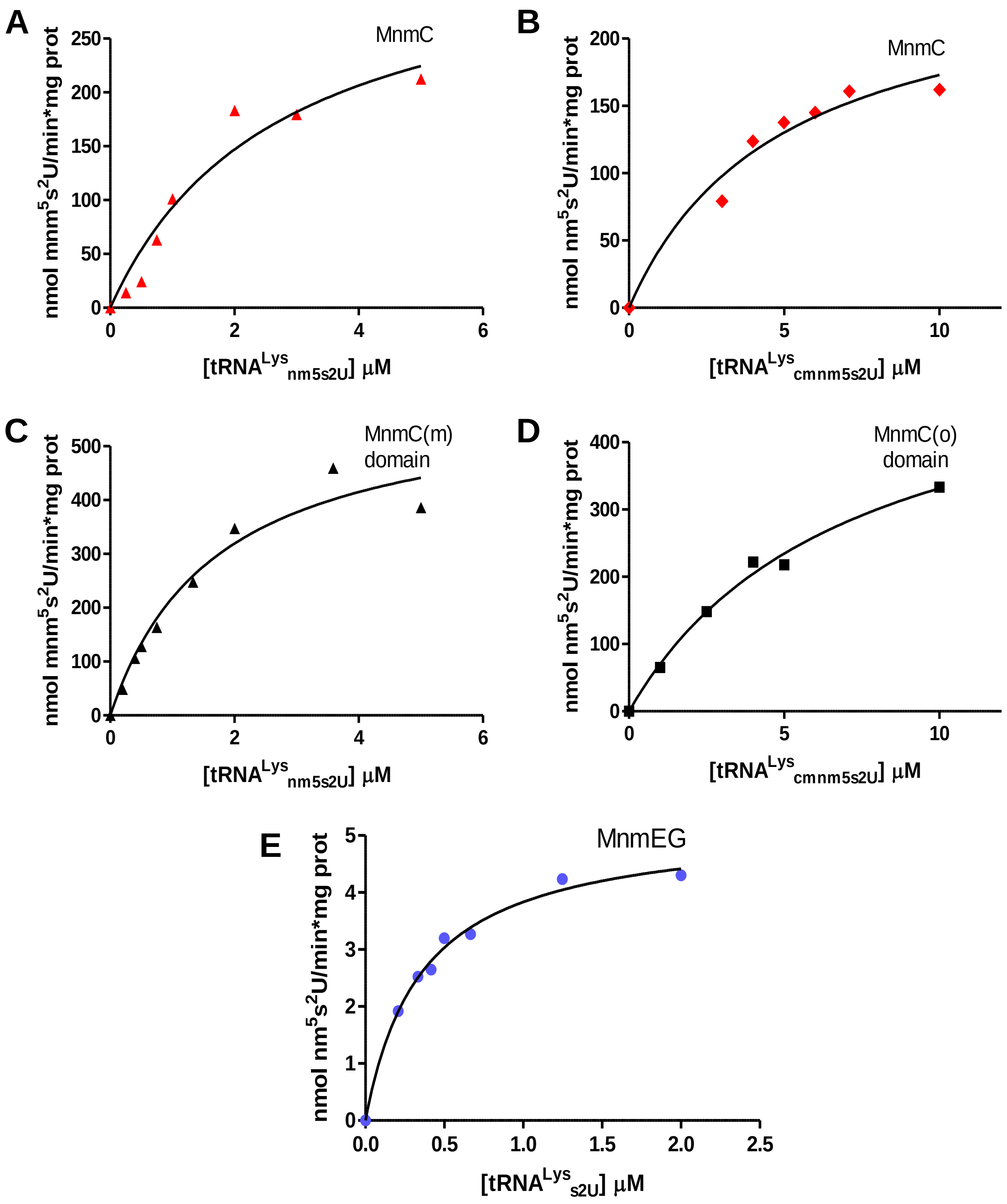

Supplementary Figure S3. Michaelis Menten plots for the MnmC and MnmEG reactions. (A and B) Michaelis-Menten plot for the SAM-dependent $nm^5s^2U \rightarrow mnm^5s^2U$ methylation (A) and the FAD-dependent $cmnm^5s^2U \rightarrow nm^5s^2U$ oxidoreduction reaction (B) carried out by the full MnmC protein. (C and D) Michaelis-Menten plot for the SAM-dependent $nm^5s^2U \rightarrow mnm^5s^2U$ methylation (C) and the FAD-dependent $cmnm^5s^2U \rightarrow nm^5s^2U$ oxidoreduction reaction (D) carried out by the separate MnmC(o) and MnmC(m) domains, respectively. (E) Michaelis-Menten plot for the ammonium-dependent $s^2U \rightarrow nm^5s^2U$ reaction carried out by MnmEG. A representative plot is shown in each panel. All reactions were performed using Chi-$tRNA^{Lys}_{mnm5s2UUU}$ as a substrate.

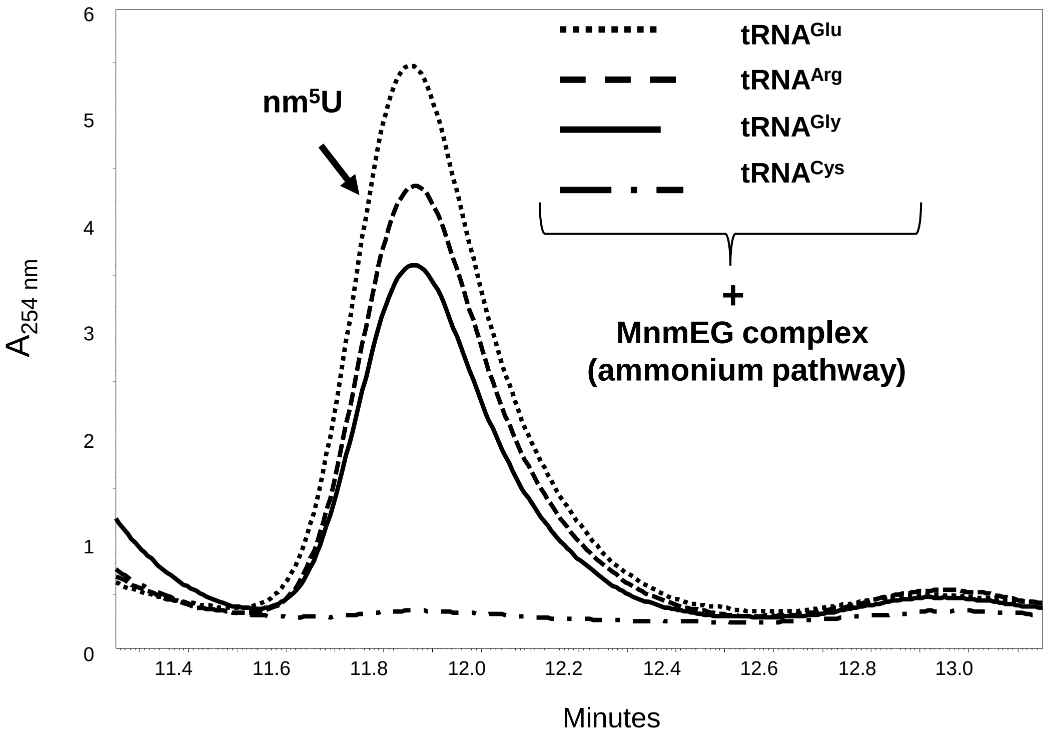

Supplementary Figure S4. MnmEG modifies the tRNA substrates through the ammonium pathway *in vitro*. In addition to $tRNA^{Lys}_{mnm5s2UUU}$, $tRNA^{Gln}_{cmnm5s2UUG}$, and $tRNA^{Leu}_{cmnm5UmAA}$ (see Figure 6), the MnmEG complex modifies $tRNA^{Glu}_{mnm5s2UUC}$, $tRNA^{Arg}_{mnm5UCU}$, and $tRNA^{Gly}_{mnm5UCC}$ through the ammonium pathway *in vitro*. $tRNA^{Cys}_{GCA}$ was used as a negative control. Reactions were performed on *in vitro*-synthesized tRNAs.

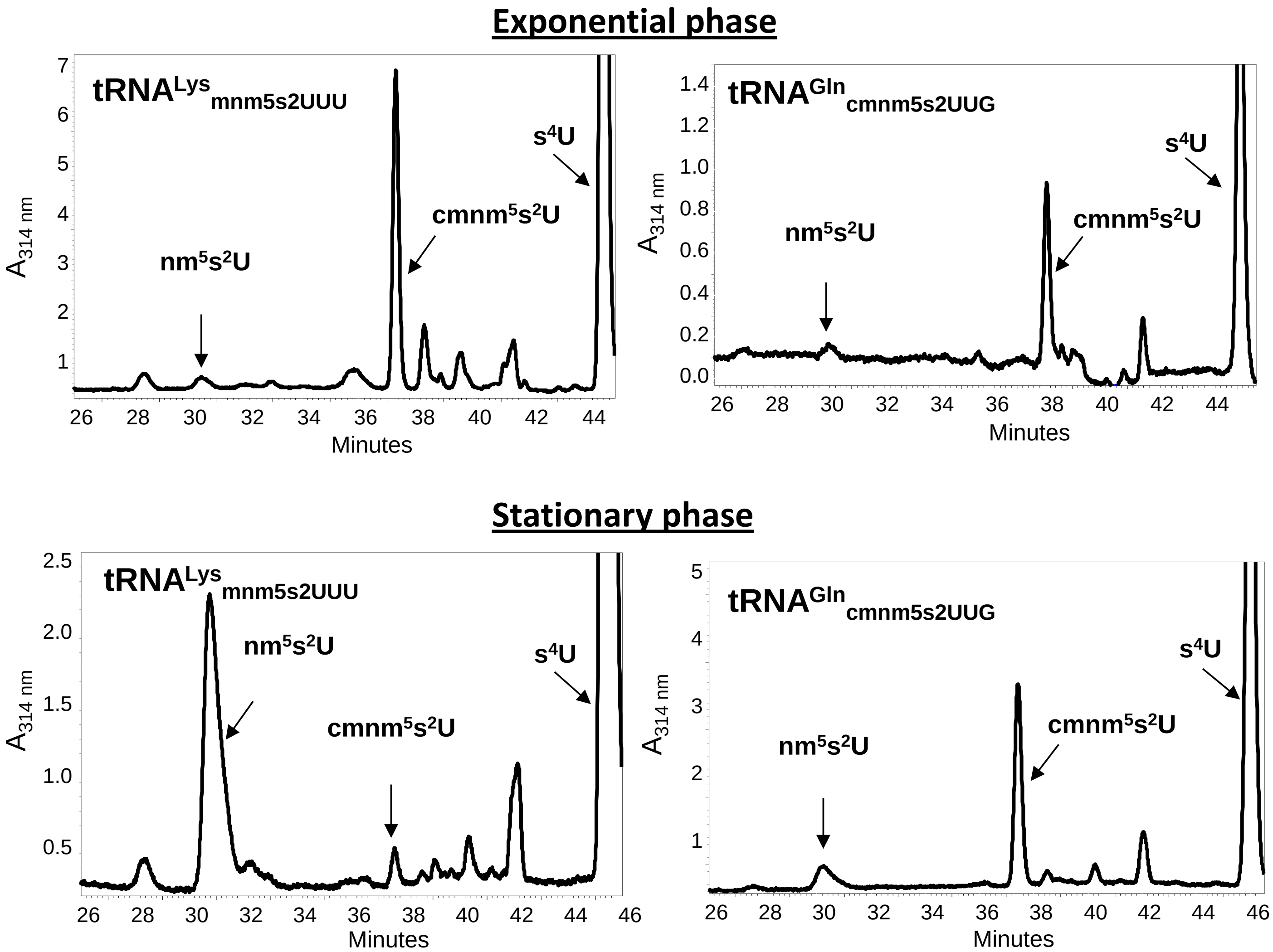

Supplementary Figure S5. HPLC analysis of native $tRNA^{Lys}_{mnm5s2UUU}$ and $tRNA^{Gln}_{cmnm5s2UUG}$. tRNAs were purified from strain IC6010 (*ΔmnmC*) at exponential (top) and stationary (bottom) phase.
